# A new framework for understanding stress and disease: the developmental model of stress as applied to multiple sclerosis

**DOI:** 10.3389/fnint.2024.1365672

**Published:** 2024-06-17

**Authors:** Michelle Fauver, Eva M. Clark, Carolyn E. Schwartz

**Affiliations:** ^1^Integral Health Program, California Institute for Human Science, Encinitas, CA, United States; ^2^MIND based Healing, Santa Cruz, CA, United States; ^3^DeltaQuest Foundation, Inc., Concord, MA, United States; ^4^Department of Medicine and Departments of Orthopaedic Surgery, Tufts University Medical School, Boston, MA, United States

**Keywords:** multiple sclerosis, stress, stress measurement, developmental psychology, core beliefs, cognitive schema, diathesis-stress model, pilot study

## Abstract

This paper proposes a new model of stress that integrates earlier models and adds insights from developmental psychology. Previous models describe the behavioral and physical effects of stress events, but have not explained the translation of experiences into stress itself. The Developmental Model of Stress shows how psychosocial developmental challenges in childhood create persistent negative beliefs and behaviors that increase threat perception and maladaptive stress responses. These developmental challenges produce early psychological and physiological predispositions for increased stress responses over time. Ongoing stress leads to dysregulation of physical stress-response systems (allostatic load), which is associated with multiple diseases. High allostatic load provides the necessary preconditions for the diathesis-stress model, which says the addition of an acute stressor to a weakened or predisposed system can lead to disease development. The paper also documents the evolving measurement of stress to better understand the stress-disease relationship, helping to resolve conflicting results between studies. The Developmental Model of Stress was combined with clinician insight and patient reports to build an integrative framework for understanding the role of stress in the development and progression of multiple sclerosis (MS). It includes the first mapping of maladaptive beliefs and behaviors arising from developmental challenges that are common to people with MS. An initial comparison shows these may be distinct from those of people with other chronic diseases. These beliefs and behaviors form the predisposing factors and contribute to the triggering factors, which are the acute stressors triggering disease onset. These often took two forms, a prolonged incident experienced as feeling trapped or stuck, and threat of a breach in a relationship. The reinforcing factors add the stress of a chronic disease with a poor prognosis and seemingly random symptom fluctuation, still managed with the same beliefs and behaviors developed in childhood, increasing physiological dysregulation and symptom severity. A pilot study is described in which these three categories of stress factors in MS were explicitly addressed. This study noted clinically important improvements in physical and mental well-being, providing preliminary support for the Developmental Model. Future research might expand on the pilot using a more robust sample and design.

## Introduction

People with multiple sclerosis (MS) consistently report distinct types of stressful events prior to the onset of disease (Mei-Tal et al., 1970). They believe there is a relationship between stress and their symptoms (Mohr and Bhattarai, 2018). Almost 150 years ago, psychiatrist and neurologist J. M. Charcot, who named and defined MS, was the first to note a relationship between stress and MS. He described MS onset as a consequence of grief, vexation, and adverse changes in social circumstances (Charcot, 1879). A clinician with 10 years of experience working with people with MS recognized some common patterns in how they perceive and respond to events, with particular types of stress events preceding disease onset and symptom worsening. These patterns, she found, are rooted in beliefs and social strategies formed in childhood that increase stress through the people’s lives.

The challenge in presenting this model is the empirical literature on stress and MS does not include the types of stress observed. This absence could explain why studies on the relationship between stress and MS yield inconsistent results (Jiang et al., 2022). They look for correlations using measures of stress that are at best only tangentially related to patient reports and clinical observations. This gap in the literature led to a thorough examination of how stress is measured, which then led to an even deeper inquiry into the evolution of stress models. Current models of stress and disease typically look at traumatic or major stressful events. They describe the behavioral and physiological effects of stress, but fail to explain the psychological process by which stress events lead to stress responses. They do not include the types of stressors and stress responses reported by people with MS.

In order to document and study the model of stress and MS formed through direct engagement with people with MS, the authors had to create a new model of stress and disease. The first half of this paper presents the resulting Developmental Model of Stress, exclusively using evidence in the available literature. This section shows the evolution of stress models and adds the psychological dimension that translates experiences into stress, briefly introducing how the Developmental Model of Stress could be applied to MS. Then it looks at the evolution of stress measurement in response to the models, using examples drawn from the MS literature to show the general principles. Finally, it details the relevant parts of developmental psychology that produce the lifelong patterns of maladaptive beliefs and behaviors that create stress, along with the early and ongoing physiological stress responses that contribute to disease development.

The second half of the paper applies the Developmental Model of Stress in its description of a new model of stress and MS. This model of stress and MS synthesizes converging evidence from clinical insights, patient reports, and medical and psychological literature into a coherent narrative. The original formulation of evidence-based medicine called for the balanced integration of empirical research and clinician insight (Sackett, 1997), while patient-centered medicine listens carefully to the person (Stewart et al., 2024). Combining science, clinical experience, and patient report is fundamental to Western medicine, as expressed by Osler (1892). This section provides an initial framework for identifying specific types of stress commonly found among people with MS. The description of this comprehensive model will include clinician and patient voices combined with the literature to support more complete understanding of how the model might appear from each of these three perspectives. The model could lead to earlier identification of people at risk for developing MS and a broader range of better-targeted therapies.

## The developmental model of stress

### Evolution of stress models

Stress is often thought of in terms of the original Stressor-Stress Response Model put forth by Selye (1936). A threat to the organism’s integrity, the stressor, occurs and elicits an automatic stress response in the hypothalamic–pituitary–adrenal axis. This fight or flight response to stress (Cannon, 1915; Cannon, 1929) is designed to marshal the body’s resources for managing the threat and returning to homeostasis. The stressor-stress response model is suited to managing discrete, short-term threats, after which the chemical response rapidly dissipates. With prolonged stress, the body enters Selye’s third stage, exhaustion, when the stress response itself creates problems in the body.

Lazarus’s Cognitive Model of Stress (Lazarus et al., 1952) introduced the role of appraisal. If the threat is appraised as larger, more imminent, or more dangerous, then the body activates a larger stress response. If the person is unconscious and unable to cognitively appraise the threat, no stress response occurs (Symington et al., 1955). The cognitive model expanded the previous stressor-stress response model to include (1) an internal or external stressful event, (2) conscious or unconscious evaluation of the event, (3) physical or mental coping processes, and (4) the stress reaction, a complex combination of physical and mental responses (Lazarus, 1993).

Engel’s Biopsychosocial Model of Medicine (Engel, 1977) added needed nuance to the cognitive model of stress, recognizing that all four steps of the cognitive model are responsive to physical, psychological, and social influences. The understanding of stress becomes richer by including the often-conflicting roles of emotions, meaning-making, biochemistry, social influences, beliefs, preferred coping mechanisms, and temperamental characteristics (Sapolsky, 2004; Surachman and Almeida, 2018). Each of these aspects influence each other, creating feedback patterns that can impact physical and mental stress responses (Oken et al., 2015). Importantly it introduced the role of psychological and social stressors. Stressors no longer required an outside event and could be self-created through sensitized thoughts and beliefs (Lazarus, 1999). Interpersonal stressors, especially those during the early developmental period of life, gained increasing attention (Pynoos et al., 1999), leading to the recognition of complex forms of response to stress. The freeze response (i.e., stress-induced immobility) was recognized as active in humans and not just other animals (Barlow, 2004). When stress researchers finally started including women in their studies, they identified another common but previously unrecognized form of stress response, “tend and befriend” (Taylor et al., 2000), referring to responding to stress by tending to and seeking connection with others. This response is most common among women and children, and most often occurs in response to interpersonal stressors. These added complexities made it difficult to develop a unifying model of stress and disease that encompasses everything from cellular dynamics to social influences.

Miller et al.’s Biological Embedding of Childhood Adversity Model (Miller et al., 2011) is one such comprehensive model. It describes the experiences of early childhood as laying the biological and behavioral foundations for future stress-related responses that are predictive of disease development. Among its key features is the life-long influence of beliefs and behaviors adopted during childhood on each of the cognitive model’s four steps.

This paper proposes a Developmental Model of Stress. It adds the findings of psychology in Engel’s biopsychosocial model (Engel, 1977) to Miller et al.’s Biological Embedding Model (Miller et al., 2011). Developmental psychology documents the dynamics of core belief formation during early childhood, and especially how the beliefs are influenced by the parent–child relationship. These core beliefs serve as the interpretive framework that guides the child’s perception of and response to environmental conditions. The Developmental Model of Stress begins with the adoption of maladaptive beliefs about self and others due to challenging interpersonal relationships during infancy and early childhood. These problematic beliefs create the conditions for distorted threat perception and limited coping responses through later childhood and into adulthood, leading to increasing levels of ongoing stress. The pattern of constant and growing stress, together with the biological changes in response to stress, creates the physical and psychological preconditions for the addition of a new stressor to overload the stress response systems and trigger the development of a physical disease. This is consistent with the diathesis-stress model of disease onset.

**Figure 1:**
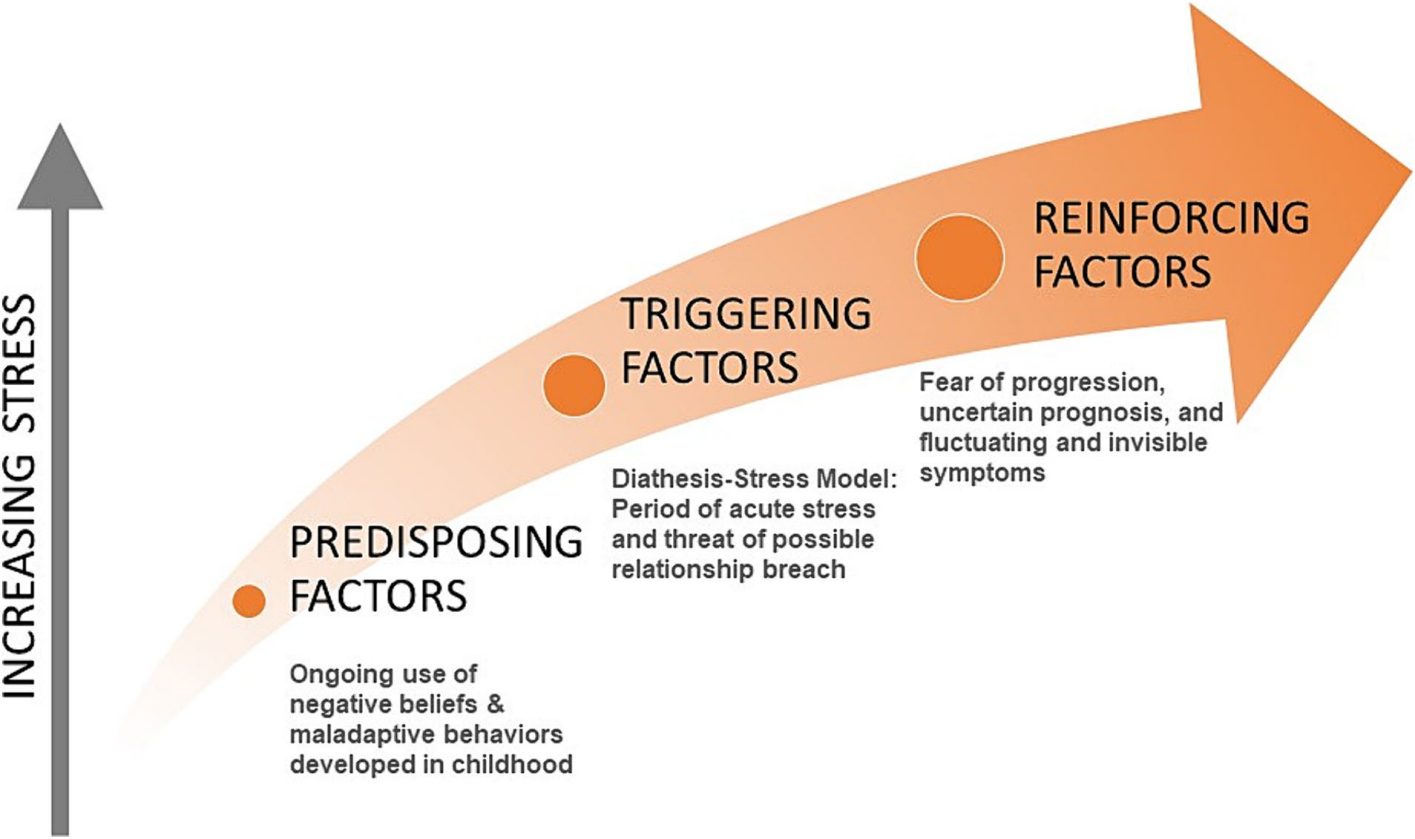
Categories of stress in people with MS.

For people with MS, the cause of overload may take the form of a period of acute stress and a threat of a relationship breach for which the person’s beliefs and coping skills are insufficient, leaving them feeling trapped or stuck and eliciting the freeze response. This leads to the first physical symptoms of MS, such as paralysis, numbing, and optic neuritis, and the consequent diagnosis. The diagnosis, with its poor prognosis and subsequent worsening of symptoms, introduces a new set of stressors which they attempt to manage with the same set of maladaptive beliefs and behaviors, adding to the mental and physical stress loads and contributing to disease progression (Figure 1).

### Evolution of stress measurement

As shown by the evolution of stress models, stressful events do not equate with stress response. Rather it is the appraisal of and response to events, informed by the person’s core beliefs, that leads to the experience of stress and its mental and physical consequences. Yet much of the research on stress and disease still uses counts of events or types of events based on the idea of cumulative risk, which assumes that more events implies more effects (Evans et al., 2013; McLaughlin and Sheridan, 2016). Studies based on counts of events must be interpreted with caution due to the presence of so many mediating and moderating factors not considered in the measures.

Moving from the stressor-stress response model to the cognitive model of stress introduces multiple appraisal, cognitive, and emotional elements. A recent systematic review looking at stress and MS reported that the included studies had inconsistent conclusions, perhaps partially due to the lack of nuanced information about stress (Polick et al., 2022). Studies that measure self-reported stress severity, or “subjective cognitive perception” (Koolhaas et al., 2011), rather than only counts of events have consistently shown significant and possibly causal relationships between stress and MS onset and progression, while those using counts of events have yielded less consistent results (Briones-Buixassa et al., 2015; Polick et al., 2023a; Shields et al., 2023).

Adding only a single variable for stress intensity, which itself is the final of four steps in the cognitive model of stress, significantly improves predictive ability (Shields et al., 2023). The Life Events and Difficulties Schedule (Brown and Harris, 1978/2001) uses a semi-structured interview format allowing greater depth of inquiry, but still results only in counts of events and a single measure of stress intensity. It has been found to predict MS onset based on perceived stress severity in the year or two prior to diagnosis (Warren et al., 1982; Grant et al., 1989).

Although it would be impossible to capture all the complexity introduced by Engel’s biopsychosocial model (Engel, 1977), a more recently developed tool combines the depth afforded by interviews with the rigor and ease of self-report instruments. The Stress and Adversity Inventory (Slavich and Shields, 2018) measures relevant biopsychosocial dimensions, such as domains of social and economic factors, acute and chronic stressors, timing of stressors, and social-psychological characteristics. Each stressor selected by the respondent is followed up with questions regarding severity, frequency, timing, and duration. Initial testing shows strong predictive ability for a range of medical diseases and metabolic functions, and is particularly effective in predicting autoimmune diseases. It captures some of the effects of stress as well, such as mood disorders and positive and negative affect, but does not address the beliefs that give rise to these effects or the coping behaviors that ensue. The Life Events and Difficulties Schedule (Brown and Harris, 1978/2001) and the Stress and Adversity Inventory are the two approaches recommended as current best practices for measuring stress in health research (Crosswell and Lockwood, 2020).

The two measures recommended above, and especially the Stress and Adversity Inventory with its broader scope and greater detail, effectively measure some types of stressful events and the emotional response to those events. They do not, however, capture the psychological dimension of why the events were experienced as stressful, nor the seemingly smaller events that give rise to stress. Automatic thoughts and expectations, along with coping strategies provide a more complete understanding of what leads to the experience of stress. These arise from core beliefs developed in childhood, which are usually modified through life experience.

There are many self-report instruments to identify core beliefs, though they may not be measuring what they intend. Core beliefs are typically unconscious and not available for recall when responding to test questions (Beck, 2020). In contrast, automatic thoughts and expectations, which result from core beliefs, are available for recall (Beck, 2020). The scales used to measure core beliefs are typically validated against measures of the effects of the beliefs, such as depression, eating disorders, or relationship difficulties. A review of 25 scales measuring beliefs reported that none of the scales tested their construct validity against an assessment of core beliefs uncovered directly through therapeutic exploration (Bridges and Harnish, 2010). The results on these types of measures can also be strongly influenced by the test-taker’s mood at the time of the test (Stopa and Waters, 2005). With these limitations acknowledged, the Young Schema Questionnaire-Rasch version seems a good quality scale not targeted to a particular diagnosis, so is able to capture a broader range of specific beliefs (Yalcin et al., 2022, 2023). It, like other belief scales, is subject to response bias in that people who use an avoidant coping strategy tend to underreport negative beliefs (Young, 2003).

Most core beliefs, and especially negative core beliefs, are unconscious (McBride et al., 2007; Beck, 2020). Cognitive behavioral therapy (CBT) has a variety of techniques for identifying beliefs, however they are most often used for identifying automatic thoughts or intermediate beliefs, and not the limiting, unconscious core beliefs developed in childhood. Some of these techniques are based on tools first developed in hypnotherapy (Watkins, 1971). Most therapists are not trained in these techniques, as CBT primarily focuses on present-day thoughts and feelings, leaving exploration of childhood experiences to psychoanalysts (James and Barton, 2004; Wedding and Corsini, 2018). Hypnosis provides faster and more effective access to core beliefs than does CBT (Brink, 2005; Dowd, 2005). Hypnotherapy and neurolinguistic programming techniques allow rapid access to the unconscious patterns of belief and behavior, making them the preferred method for identifying core beliefs (Dilts, 1990; Davis and Davis, 1991; Lucas, 2007).

### Developmental psychology and stress

Stressful events such as adverse childhood experiences often lead to problems with physical and mental health in adulthood (Felitti et al., 1998; Berens et al., 2017; Polick et al., 2023b). Research on stress and disease has for the most part focused on stress events, especially traumatic events, and biological responses to stress. Many of the physiological pathways involved in translating the experience of stress into physical disease have been identified and mapped (McEwen, 1998; Miller et al., 2009; McEwen, 2015). The events have also been shown to be associated with maladaptive beliefs and behaviors (Miller et al., 2011), but the pathway for translating stress-related experiences into the types of problematic threat assessment and coping behaviors observed has not commonly been included in the discussion (Friedman, 2008).

Different types of early life adversity lead to different types of psychological and physiological responses. Preliminary evidence suggests these differences might usefully predict a variety of diseases (Berman et al., 2022). Studies using counts of adverse childhood experiences can still be informative when findings are broken down by types of stressors. Using counts of adverse childhood experiences, a large-scale cohort study (Riise et al., 2011) found no link between later diagnosis with MS and severe physical abuse or forced sexual activity during childhood and adolescence, while another count-based study found childhood experiences such as household dysfunction and neglect were linked to MS onset (Shaw et al., 2017). A third study looking at counts of childhood stressors found no overall statistically significant correlation with later MS development, but when broken down by type of stressor again showed emotional stressors were more correlated with MS onset than were physical stressors (Briones-Buixassa et al., 2017). A fourth study looking at the relationship between MS fatigue symptoms and types of childhood adversities found childhood emotional neglect and emotional abuse were the strongest predictors of fatigue (Pust et al., 2020).

A meta-analysis of different kinds of childhood adverse events and their association with negative core beliefs (Pilkington et al., 2021) found the strongest predictors of the child developing negative core beliefs were all emotional or interpersonal in nature. It also showed physical stressors influence the development of negative beliefs very little. Negative core beliefs lead to heightened threat perception and influence coping responses (Rakhshani and Furr, 2021). Children with negative core beliefs then report a higher number of adverse events through their later childhood and teen years (Rakhshani and Furr, 2021).

#### Developmental stress

The World Health Organization defines stress as “a state of worry or mental tension caused by a difficult situation” (WHO, 2023). Stress is first and foremost a psychological state. It results from the common, ordinary, everyday way we think of ourselves and the world. It is not dependent on outside events, minor or major, to create. One person may feel relaxed sitting in traffic while another may escalate more easily. The psychological state is what gives rise to the physical and psychological responses to stress. As the evolving models of stress show, individual agency links the stressor and the stress response. But what gives rise to the psychological experience of stress? Existing research points in an interesting direction.

Developmental psychology shows the nature of this agency during early childhood and how it later influences stress experiences. Erikson’s Psychosocial Development Theory describes the impact of social experiences from infancy onward through eight stages over the lifespan, with each stage presenting challenges and opportunities for growth (Erikson, 1950; Erikson, 1959; Erikson, 1968). Gilligan’s research with women suggested the need for an expansion of Erikson’s model (Gilligan, 1982). Franz and White integrated the two perspectives into an eight-stage model incorporating both Erickson’s emphasis on separation and individuation and Gilligan’s emphasis on care and relationship, finding both aspects are important for healthy development in both sexes (Franz and White, 1985). When these early developmental stages are not successfully completed, the child can form unhealthy beliefs about themselves and the world around them (Marcia and Josselson, 2013). Extensive empirical research supports these models (Vaillant and Milofsky, 1980; Bosma and Kunnen, 2001), though with some flexibility in the ages associated with each stage (Vaillant and Milofsky, 1980).

Each child is born with a distinct temperament, with some traits inherited and some unique to the individual (Saudino, 2005). Temperament is defined as the innate attitudes and behaviors a child has for experiencing and relating to the world around them (Friedman and Schustack, 2007). These traits are generally stable across the lifetime (Thomas and Chess, 1977), and include basic emotional needs and patterns of expression (Russell, 2003; Panksepp, 2005).

The child may change the expression of their temperament through learning and conditioning in response to caregivers. Goodness of fit refers to the degree of congruence between the personality and preferences of a parent with the temperament of the child (Chess and Thomas, 1991). A close-enough matching supports the child’s psychological and social development and creates a positive relationship with the parent, while poor matching may introduce developmental delays and adversely affect the nature and quality of the relationship with the parent (Newland and Crnic, 2017). Warm and attentive care from parents that is responsive to the child’s needs can foster the development of positive core beliefs about self and others, while inconsistent or ambivalent attention from parents can foster the development of negative core beliefs about self and others (Wellman and Gelman, 1992; Wearden et al., 2008).

The relational difficulties that form the roots of limiting beliefs and result in maladaptive behaviors need not be traumatic or abusive in nature. Infants are extremely social beings, reliant on their caregivers for emotional feedback and learning. For example, when a mother or father holds their baby but does not mirror back the baby’s facial expressions, the baby goes into a state of extreme distress (Tronick et al., 1978; Mesman et al., 2009). The overwhelm of the infant’s central nervous system can be so severe the child may physically collapse (Tronick et al., 1978; Mesman et al., 2009). Even when mirroring returns, the infant’s happiness response is somewhat reduced. This shows how even small mismatches can lead to formative changes in the child’s emotional development. There are many possible reasons a parent may not be able to be emotionally present to a child, but if it persists for more than short periods it can lead to impairment of the child’s emotional and social development (Bornstein, 2014).

Negative core beliefs, also referred to as maladaptive cognitive schemas or early maladaptive schemas (Young et al., 2003), create the child’s basic “rules for living.” These negative beliefs are directly associated with not successfully completing developmental stages due to the nature of the parent–child relationship (Thimm, 2010). They form the person’s identity and patterns of relationship with self, others, and the world. Normative development shows the core beliefs formed in early childhood through the parent–child relationship typically adjust and modify in response to later engagement with peers during adolescence. Negative core beliefs formed in early childhood are more persistent, remaining in place despite peer influence (McCarthy and Lumley, 2012). They remain fixed and outside the person’s awareness well into adulthood (Tang et al., 2020).

All input to the person’s awareness is filtered through these beliefs. Negative core beliefs lead to distorted perception of outside events that reinforce these negative beliefs. The person is more likely to recognize, interpret, or recall information that matches their negative beliefs and ignore or not even perceive information contrary to their beliefs (Ball, 2007; Beck, 2020). This unconscious reinforcement causes the maladaptive beliefs to become resistant to change later in life (Riso and McBride, 2007; Curran et al., 2021).

#### Psychological effects of developmental stress

Application of the Developmental Model of Stress includes the role of negative beliefs formed in early childhood leading to characteristic experiences of stress. These may vary with different diseases. A broad range of psychological factors contribute to MS onset and symptom severity that are distinct from people who do not have MS (Liu et al., 2009). This includes aspects of mood, emotions, threat expectations, coping responses, and social relationships (Liu et al., 2009). All of these can be related to negative core beliefs.

Early adversity may lead the child to develop maladaptive beliefs and behaviors that increase the frequency and types of stressor perception (McLaughlin et al., 2014). The dysfunctional patterns of threat assessment occur both consciously and unconsciously, continuing into adulthood (Finlay et al., 2022). The brain’s pathways for threat detection become increasingly over-sensitized through ongoing perception of threats, leading to even more detection of stress events (McEwen, 2017). This pattern of constant alertness combined with distorted brain function can lead to increased stress even at rest (Lanius et al., 2017).

The negative core beliefs developed in childhood have been found to correlate with later distortions in mood and neurocognitive development (Beck, 2008). A disengaged or overly-controlling parent can lead the child to develop negative beliefs about themselves (Garber and Flynn, 2001). These negative self-concepts may be adjusted during teen years if parents change their engagement style, but the negative self-beliefs tend to continue into adulthood (McArthur et al., 2019). Negative core beliefs also lead to problematic relationships with others, and these patterns too continue into adulthood (Waldinger et al., 2002; Simard et al., 2011). They often lead to a sense of constant and generalized anxiety (Koerner et al., 2015), which increases the experience of stress and continues into adulthood (Tariq et al., 2021). They predispose the person to have difficulty recognizing and managing their own emotions, especially when the beliefs were related to unmet needs for attachment and autonomy (Pilkington et al., 2024). Further, they produce lifelong maladaptive coping patterns (Curran et al., 2021).

This combination of interacting maladaptive beliefs and behaviors lead to complex and compounding challenges in self-identification and relationships with others. The initial small-scale parent–child difficulties during early childhood, when repeated over time, lead to developmental disruptions in the child’s self-and social awareness, emotional attachments, cognition, and emotional awareness (Cruz et al., 2022). These dysfunctional developmental patterns create a self-perpetuating cycle of ever-increasing emotional tension as the child tries to apply them in the larger world, leading to increasingly complex patterns of maladaptive beliefs and behaviors (Herman, 1992). These more complex patterns of stress perception and response share many features with post-traumatic stress disorder (Chiu et al., 2023). The more complex stress-based psychological difficulties may first show themselves in late teen years or early adulthood (Cloitre et al., 2009), yet are most often found among people who experienced interpersonal developmental challenges (Giourou et al., 2018). These patterns of complex responses have been found to mediate the relationship between childhood developmental challenges and disease development (Ho et al., 2021).

#### Physiological effects of developmental stress

Most research on the physiological effects of stress look at adults. While this approach makes sense because most stress-related diseases are typically found in adults, the physiological precursors can be found in childhood just as for the psychological precursors. Poor quality parent–child interactions have been shown to increase childhood cortisol levels during the first 5 years of life (Moffett et al., 2007). A longitudinal study found it also predicted decreased brain volume in both later childhood and mid-to late-adolescence, particularly in the emotion and executive centers, to a similar degree as found in children who had experienced severe traumatic events (Tyborowska et al., 2018).

Contrary to early thinking that activation of the hypothalamic–pituitary–adrenal axis occurred in response to threats to biological integrity, the adrenal response is almost exclusively triggered in response to psychological stressors and only barely responds to physical stress (Mason et al., 1976). No research has yet been done with humans to distinguish different types of physiological responses to different types of early life stressors. Animal models suggest environmental, physical, and early-life relational stressors all activate the hypothalamic–pituitary–adrenal axis to varying degrees, and increase its activation with repeated exposures, but only early-life relational stressors reduce the ability to down-regulate the stress response (Kuhlman et al., 2017).

The original article on Miller’s Biological Embedding model provides detailed reporting of the physical changes in childhood observed in response to stress (Miller et al., 2011). These include changes in the development and function of the endocrine and central nervous systems, all the way down to cellular metabolic functions. These changes program the body for heightened reactiveness to stress and reduced immune functions. The paper also proposed some pathways by which this dysregulation occurs that have since been supported (Enoch, 2011; Dye, 2018), showing changes even down to what parts of DNA get expressed (Bigio et al., 2023; Zhou and Ryan, 2023).

The above physiological stress responses in childhood create the early predispositions for greater physiological dysregulation later. Stress is causally associated with changes in chemical balance, metabolism, organ function, and tissue structure. When stress become perseverative, it causes ongoing and lasting physical changes (Berens et al., 2017). McEwen’s concept of *allostatic load* represents the body’s multi-system adaptation to chronic stress (McEwen and Stellar, 1993). Allostatic overload is when even this complex compensatory system can no longer manage demands, leading to the severe dysregulation of metabolic, cardiovascular, and immune functions, along with the sympathetic and parasympathetic nervous systems, and changes in the structure and function of the brain (McEwen, 2017). This severe dysregulation of organismic systems sets the stage for an acute stressor to trigger the onset of a physical disease, following the diathesis-stress model of disease development (Lazarus, 1993; Monroe and Cummins, 2014).

The evidence for the role of stress in multiple sclerosis is clear. High allostatic load is common in people with MS (Waliszewska-Prosół et al., 2022). It has been shown to cause most of the symptoms of MS. It can cause prolonged inflammation (Gold et al., 2005; Lenart-Bugla et al., 2022), neurodegeneration (Harnett et al., 2020), and motor dysfunction (Snell et al., 2022); and increase disability (Gold et al., 2005). High stress and allostatic load can also lead to chronic pain and emotional dysregulation (Nakamoto and Tokuyama, 2023). Linking this stress-related allostatic load to psychological processes can help better inform the biological development and progression of MS.

This paper has so far introduced a model of stress and disease that moves beyond original formulations identifying adverse or traumatic events as the cause of stress, and adds the role of childhood development as a critical factor in understanding stress through adulthood. The maladaptive beliefs and behaviors originating from developmental challenges are robust, remaining essentially unchanged through life unless directly named and addressed. These unconscious beliefs and behaviors profoundly influence stress perception and response. The remaining sections of the paper will describe how these principles can be applied to the study of stress and MS.

## The developmental model of stress applied to MS

The Developmental Model of Stress proposed here provides a tool for further inquiry into the stress-MS relationship. The following model of stress and MS includes three categories of stress experienced by people with MS over their lifetime that contribute to the physical expression and progression of the disease. These categories include (1) predisposing factors developed in childhood, (2) triggering factors preceding disease onset, and (3) reinforcing factors that continue during the course of the disease. These factors were identified through clinical observation, patient reports, and concurrent examination of the literature. These three perspectives are illustrated in Tables 1–3. Clinician Voice represents clinical insights gained by a certified clinical hypnotherapist while working with people with MS. The Patient Voice comments are in italics to indicate direct quotes recorded during the hypnotherapy sessions illustrating the factors identified in the Clinician Voice to the left. The Literature Voice does not reflect each of the points on the left, but rather describes the overall process for each of the three categories of factors based in medical and psychological literature.

**Table 1:** Model summary – predisposing factors in three perspectives.

Predisposing factors	From age 0 until about 2 years prior to diagnosis	
Clinician voice	Patient voice	Literature voice
Developmental challenges in early childhood produce two broad categories of problematic thinking: Guilt and shame about their own emotional needs and wants. The need to conform to what is expected rather than expressing individual preference and autonomy. These result in a set of five negative beliefs and behaviors commonly observed in people with MS. **Emotionally Parentified Child** Poor boundary formation. Managing parent’s emotions. Not learning own needs. **I am Not Enough** Inability to know when enough effort has been expended. Cannot stop and recover, need to keep going. **Severe Inner Critic** Can be self-loathing. Accompanied by guilt, shame, and regret. **External Self-Worth** Worth based on external achievements. Strong need to be accepted by others. **Rigid Decision-Making** Based on what is right, perfect, or expected. Based on safety. Fear of failure.	*I would describe my father as strict. It was important we did things “right.”* *Both my parents worked and were busy.* *I was alone in my room for hours.* *My mother was strong-willed, and not at all tender.* *I learned early on to just do things myself.* *I can’t be happy if someone else, especially family, is upset. I am miserable.* *I always felt I had to protect my mother.* *I try to fix things for people, even if that means putting off my own stuff.* *I never think I’ve done enough, no matter how hard I work.* *I have an internal pusher. I cannot be lazy.* *For years, I hated myself.* *Regret about the things I did in my early 20’s still eats at me.* *I am a people pleaser. I need to be accepted by everyone.* *If I fail, no one will love me.* *I do everything the right way, including scooping peanut butter from the jar.* *I chose law as my career because that was the sensible thing to do.* *I’d rather not do it if I think I can fail.*	Psychosocial developmental models show the importance of learning both independence and positive relationships through healthy parent–child interactions. When positive parental emotional support is lacking the child may develop negative core beliefs about themself and others. Core beliefs shape stress perception and response. Early interpersonal stressors create immediate physical stress responses in childhood and the physiological predispositions for larger inflammatory, immune system, and neurological responses later. Negative core beliefs are perseverative, lasting through adolescence when normative beliefs typically adjust in response to peer feedback. This leads to distorted threat perception, increasing the number of events eliciting the stress response through teen years, and building allostatic load. Maladaptive coping behaviors rooted in early coping strategies become less capable of managing stressful situations. This leads to increasing distress, particularly in social and interpersonal and relationships. The increased distress produces many of the psychological and physiological symptoms associated with post-traumatic stress disorder (PTSD), without the presence of specific traumatic events and while seeming to be highly functional.

Clinician Voice represents clinical insights gained by a certified clinical hypnotherapist while working with people with MS. Patient Voice comments are in italics to indicate direct quotes illustrating the factors identified in the Clinic Voice to the left. The Literature Voice does not reflect each of the points on the left, but rather describes the overall process for each of the three categories of factors based in medical and psychological literature.

**Table 2:** Model summary – triggering factors in three perspectives.

Triggering factors	During the 2 years prior to diagnosis	
Clinician voice	Patient voice	Literature voice
People with MS almost always experienced a prolonged period of stress accompanied by a relational separation conflict. First symptoms may occur up to 6 months after conflict resolution. **Prolonged Period of Stress Prior to Onset** Experienced as feeling of being trapped or stuck. **Culminating Incident** Typically experienced as a relational separation conflict.	*There was an economic downturn, but I was determined to increase my sales that year. I never worked so hard in my life.* *Right when I was getting my life back together, I noticed my foot dragging.* *I want to get out of it, but I do not know how, so I push forward.* *I was so stressed with work but I could not leave or I’d lose my work visa.* *I stayed for far too long until I realized that if I stayed, he’d kill me.* *When I shared my distress with my mother she told me “How could you say that? You should be grateful you have this.” I was in shock.*	Ongoing stress can increase mood disorders and emotional problems, both observed in people with MS prior to diagnosis. The diathesis-stress model from medicine and psychology shows ongoing stress leads to heightened stress response. Adding an acute stressor can trigger the onset of disease. Interpersonal relationship stressors similar to the types found in childhood may lead to unconscious flashbacks similar to those in PTSD. Feeling trapped in a stressful situation, especially after ongoing and acute stressors, may lead to the physiological freeze response to stress.

Clinician Voice represents clinical insights gained by a certified clinical hypnotherapist while working with people with MS. Patient Voice comments are in italics to indicate direct quotes illustrating the factors identified in the Clinic Voice to the left. The Literature Voice does not reflect each of the points on the left, but rather describes the overall process for each of the three categories of factors based in medical and psychological literature.

**Table 3:** Model summary – reinforcing factors in three perspectives.

Reinforcing factors	After diagnosis	
Clinician voice	Patient voice	Literature voice
Being diagnosed with a chronic progressive disease with no cure, usually as a young adult, is highly disruptive to identity, behavior, and future plans. **Response to Diagnosis** Highly acute distress. Isolating. Suicidal ideation. **Beliefs about MS** Expectation of progression. Any symptom change is associated with progression. **Living with Symptoms of MS** Symptom variability decreases activity predictability. Symptom invisibility. Stressors similar to Triggering Factors cause symptom worsening.	*Who is going to want to be with a sack of potatoes?* *I am a caregiver turned reluctant patient.* *I thought my life was over.* *I try not to burden my husband with my symptoms and feelings.* *Right after diagnosis, I kept having thoughts about driving off the road.* *I was told by my neurologist that I only had a few years left before MS would take my faculties away.* *After the shot, my symptoms got worse for four days. I could barely walk. I was sure I would not recover.* *Sometimes I feel almost normal and other days I can barely lift myself out of bed and have to cancel all my plans.* *My colleagues at work think I’m just trying to get out of working. They don’t know I have MS.* *My friend wanted me to go shopping with her this weekend, but I really didn’t want to. I went anyway. The next morning, I could barely walk up the stairs!*	Diagnosis of MS is typically combined with a dire and mostly negative prognosis for disease progression. This can produce the nocebo response, worsening symptoms in the absence of a known noxious agent. The nocebo response is increased in people with a psychological profile commonly found in people with MS. The beliefs developed in childhood often lead people with MS to avoid thinking about the disease and to use maladaptive coping behaviors that isolate them from social support, leading to worse outcomes. Repeated exposure to social and interpersonal conditions similar to those that triggered disease onset may contribute to symptom progression.

Clinician Voice represents clinical insights gained by a certified clinical hypnotherapist while working with people with MS. Patient Voice comments are in italics to indicate direct quotes illustrating the factors identified in the Clinic Voice to the left. The Literature Voice does not reflect each of the points on the left, but rather describes the overall process for each of the three categories of factors based in medical and psychological literature.

Consistent with how this model was developed, the rest of the paper will include all three sources of information. Most of the following discussions of the identified factors will begin with how they are identified and understood clinically, then be illustrated with direct quotes from clients in italics, and then be followed by a discussion of the relevant scientific literature. This pattern allows a richer understanding of the dynamics involved and could lead to more fruitful discussions between people with MS and the people providing care.

### Predisposing factors

Clinical observation found that people with multiple sclerosis appear to have a common set of negative core beliefs and maladaptive behaviors that are distinct from those of clients with other diseases and disorders. These beliefs and behaviors were experienced by clients as fixed and defining of self, others, and the world. “*This is just who I am*” and “*Doesn’t everyone fear failure?*” were common reactions at the start of exploring these beliefs.

Two childhood developmental challenges were detected that may have contributed to the formation of the majority of the commonly observed negative core beliefs. These were the failure to have core emotional needs met and not learning to assert independence. In particular, clinical observation found clients experienced (1) guilt and shame about their own needs and wants, even to the point of not being able to recognize them, “*I don’t have needs*” and (2) the need to conform to external wishes, such as doing things the “*right way*” or what seems to be expected, rather than expressing individual preferences and autonomy.

While the parent/child interactions that created these developmental challenges were not necessarily traumatic, they were impactful. Not getting their emotional needs met could have been caused by experiences such as being an only child with busy professional parents; the mother or child being hospitalized during early childhood; or having teenage parents, a distracted grieving parent, or a worried parent. “*I was alone in my room for hours and hours*.” Additionally, adaptation to external expectations rather than developing autonomy seemed to be influenced by a strict, controlling, or corrective parent, “*My dad always said that I could do better*,” or through the child managing the parent’s emotions, taking care of them to “*keep my mother from worrying about me*.”

As described in the Developmental Model of Stress, negative beliefs and maladaptive behaviors are primarily formed through parent–child relationship challenges. Most relevant to people with MS, these challenges include parental disengagement, where a child’s emotional needs are not met, or an over-controlling parent, where autonomy is not encouraged (Garber and Flynn, 2001; Thimm, 2010). Developmental challenges that include household dysfunction and neglect have been linked to MS onset (Shaw et al., 2017). The negative beliefs and behaviors adopted during childhood are key features in future stress-related responses predictive of disease development (Miller et al., 2011).

The distortions in self-identity, behaviors, relationships, and emotions introduced through developmental challenges are wide-ranging and overlapping, influencing each of the core beliefs and behaviors named in the Predisposing Factors section below. To give an example of the complexity involved, alexithymia is a contributing factor to several of the beliefs and behaviors identified as associated with MS. Alexithymia is usually defined as difficulty in identifying and processing emotions (Sifneos, 1973), but that definition has since been expanded. The difficulty in identifying emotions in self or others is particularly high for negative emotions (Luminet and Zamariola, 2018), and those with higher levels of alexithymia may actually display higher emotional responses to events and/or use emotional responses designed to take care of others (Luminet et al., 2021). It also includes an underdeveloped sense of self, lack of an internal world, and externally oriented thinking (Berenbaum and James, 1994; Lumley et al., 2007).

Alexithymia is found in about 10% of the general population (Honkalampi et al., 2001; Franz et al., 2008) and in up to 53% of people with MS (Chalah and Ayache, 2017; Eboni et al., 2018). It can appear in early childhood as a result of interpersonal challenges during developmental stages (Berenbaum and James, 1994; Wearden et al., 2003; Aust et al., 2013; Ditzer et al., 2023). Symptoms of alexithymia in childhood may slightly reduce during adolescence (Kekkonen et al., 2021), but when they arise in response to developmental challenges they typically remain stable into adulthood (Tolmunen et al., 2011; Ditzer et al., 2023). People with MS and alexithymia show higher rates of anxiety, depression, and fatigue (Chalah and Ayache, 2017; Eboni et al., 2018; Kekkonen et al., 2021), and continuous hyperactivity of the sympathetic nervous system’s stress response functions (Chalah and Ayache, 2017). Brain studies show that people with MS have difficulty in reducing emotional reactivity, especially in response to negative emotions, and that this inability is increased for those with alexithymia (Van Assche et al., 2021).

Alexithymia can also represent an attempt to “freeze” or deny emotions in an attempt to reduce emotional distress (Chahraoui et al., 2015). People with MS and alexithymia tend to use negative coping mechanisms, resorting to self-denial and submission strategies rather than more problem-focused strategies (Yilmaz et al., 2023). This is related to the tend-and-befriend stress response, used in the hope that tending to others’ need will reduce their own distress (Taylor et al., 2000). While higher levels of alexithymia is associated with higher emotional responses designed to take care of others (Luminet et al., 2021), this does not necessarily improve relationship quality as adults with alexithymia show significant difficulties in interpersonal relationships (Koppelberg et al., 2023) that can also affect physical health (Wearden et al., 2003).

The negative beliefs resulting from developmental challenges commonly found in people with MS were grouped into five main types. Each will include examples from clinical observations illustrated with direct quotes from people with MS. Rather than include all of the multiple facets of issues related to each factor, the literature following the clinical and patient voices will highlight one expression of the problematic belief or behavior.

#### Emotionally parentified child

Most people observed had issues with personal boundaries and showed traits typical of people who in childhood were tasked with emotional caretaking of an adult. Blurring the lines of personal boundaries and roles was often subtle – taking on the role of emotional confidante to a parent, being a single parent’s companion, mediating family conflicts, or being emotionally supportive of a grieving or mentally ill parent. “*I always felt I had to protect my mother.*” This translated into a need to help significant others while not receiving such help themselves. “*I cannot ask for help, no matter how much I need it*” and feeling responsible for other’s emotional states “*I can’t be happy if you are not happy.*”

Emotional parentification means a role reversal in which the child takes responsibility for the emotional needs of a parent, rather than the parent providing for the child’s needs for attention, comfort, and guidance (Chase, 1999). This role reversal is recognized as destructive for the child and for the adult they will become (Boszormenyi-Nagy, 1973; Chase, 1999). This role reversal also contributes to building strong social skills as they learned to be attentive to others’ needs (Roling et al., 2020). In adulthood, this dynamic may show as assuming too much responsibility in relationships and hyper-attentiveness to taking care of others (Valleau et al., 1995). The need to be emotionally available for the parent when a parent is not as emotionally available for the child can lead to chronic anxiety and distress (Hooper et al., 2008; Engelhardt, 2012). While childhood parentification has not been studied in people with MS, one study found childhood experiences such as household dysfunction and neglect, which often leads to parentification, was linked to MS onset (Shaw et al., 2017). Childhood emotional parentification can be a driver for each of the other predisposing factors.

#### I am not enough

The second common trait was the inability to know when enough effort had been expended. Clinical observation found that clients were constantly “*doing 110%*” effort in projects, continuing to think about projects after delivery, “*Did I do enough?*,” and were not easily able to prioritize what needs effort and what could be done less thoroughly. This also translated into an inability to stop and recover. “*I keep thinking that I can stop when everything gets done, but there is always more to do, isn’t there?*”

Parentification in childhood, when one is ill-prepared for the responsibilities, can lead to imposter syndrome in adulthood, with the person feeling like they are never good enough (Chase, 1999; Castro et al., 2004). The adoption of a parenting role as a child can also lead to becoming a chronic doer in adulthood, constantly seeking self-worth through being of service to others (Chase, 1999). The additional work performed leads to increased emotional distress rather than to increased satisfaction (Menghini et al., 2023). Childhood emotional neglect and emotional abuse, which leads to parentification and feeling not good enough, predicted MS fatigue symptoms (Pust et al., 2020).

#### Severe inner critic

Another trait consistently found in people with MS was a severe inner critic that analyzes and criticizes what one “*should*” be doing at all times. This was experienced as constant vigilance regarding one’s actions, often accompanied by guilt and shame. Consequently, many clients still held onto regret regarding actions taken in early adulthood “*I should have known better.”* In some cases, the result was self-loathing. “*For years, I hated myself.*”

Parentification in childhood means the child puts aside and never develops their innate talents and gifts, focusing instead on meeting the parents’ needs or expectations (Hooper et al., 2014). This pattern in childhood predicts proneness to feeling guilt and shame in adulthood (Wells and Jones, 2000). While guilt is associated with a behavior, shame is associated with a negative evaluation of self (Muris and Meesters, 2014). Proneness to self-conscious emotions, such as shame, guilt, and regret, increases in association with the types of childhood developmental challenges that produce parentification (Muris and Meesters, 2014). This constant self-criticism has been shown to contribute to depression and anxiety (Zhang et al., 2019), both common in MS, and to an insecure attachment style in relationships during childhood (Kim et al., 2020) and continuing into adulthood (Rogier et al., 2023). Self-blame has been shown to decrease the quality of life in people with MS (Kołtuniuk et al., 2021). The severe inner critic leads the person to satisfy their need for approval by turning outward, toward gaining approval from others (Muris and Meesters, 2014).

#### External self-worth

Clinical observation found that self-worth was most often based on external achievements, such as the desire for yearly promotions, as well as a strong need to be accepted by others. Worth and identity seemed to be defined by one’s actions. This would cause a severe fear of failure and assumptions such as “*If I make a mistake, I am a failure*” and “*If I fail, no one will love me.*”

While a healthy sense of self-worth begins in childhood through affirmation by a parent, a healthy sense of worth in adulthood needs to be internally based rather than based on external feedback (Reitz, 2022). Continued basing of self-worth on external feedback is often linked with insecure attachment or the experience of conditional love (Park et al., 2004). Being raised by conditional, negatively-evaluating parents, as well as experiencing parentification, can lead to the interpretation of one’s worth as a person through achievement and success alone, resulting in overwhelm, pressure to consistently perform optimally and prove one’s worth, and exhaustion (Muris and Meesters, 2014). The need to prove worth through achievement is especially prevalent in girls (Herrmann et al., 2019) and women (Menghini et al., 2023), which might help explain the increased incidence of MS in women since they started entering the workforce in large numbers (Dunn et al., 2015). Taking on too much work as a means to show worth can lead to increased perceived stress and decreased quality of life (Lichtenstein et al., 2019).

#### Rigid decision-making

Lastly, a trait also common in people with MS was the criteria for decision-making. This style of decision-making is based on what is perceived as right, perfect, or expected, possibly developed in response to not feeling safe enough, feeling the need to please, or fearing rejection by a parent. “*I try to do everything the right way.*” One common request by clients with MS at the start of clinical sessions was, “*Tell me what I need to do and I will do it.”* This need to do it right or perfect, following external standards, also translated into the preference not to take action if there is a chance of failing. Though this common trait can affect small decisions, such as eating the “*right*” things, it can also negatively affect long-term decisions, such as career choice. “*I chose law because it was a sensible career*.”

As children develop, they learn to progressively transition from externally driven guidance and decision-making, to exhibiting more self-direction and autonomy (Frick and Chevalier, 2023). People with MS, however, continue to exhibit low self-directedness (Gazioglu et al., 2014). Adolescents who were parentified as children experience extreme distress when confronted with a possible breach in a relationship caused by the tension between who they experience themselves to be and what the other person wants them to be (Goldner et al., 2022). This often leads to them feeling stuck and defaulting to the other person’s needs (Goldner et al., 2022). Decision-making is already fraught with error for people with MS (Farez et al., 2014), especially when associated with ambiguous conditions or with risk (Farez et al., 2014). Alexithymia, common in MS as mentioned earlier, can cause inaccurate assessment of situations and make it difficult to make decisions or increase the likelihood of making incorrect decisions (Zhang et al., 2017). Parentification in childhood can influence the person’s choice of career, and make them more dependent on emotion-focused coping strategies (Boumans and Dorant, 2018; Roling et al., 2020). Decision-making based on perfection and elevated standards is a strong predictor of anxiety (Koerner et al., 2015), and perfectionism can cause maladaptive behaviors that contribute to MS fatigue (Magnusson et al., 1996).

#### Comparing beliefs between chronic diseases

Many studies have found correlations between specific core beliefs and the development of particular physical and mental health problems. For instance, types of core beliefs predict differences between mentally healthy and at-risk youth, and further analysis found unique negative core beliefs differentiate among types of mental illness (Cowan et al., 2019). Waller et al. found that distinct types of negative core beliefs predicted different types of eating disorders (Waller et al., 2003). Further, different types of skin disorders are associated with overlapping but distinct groups of negative core beliefs (Mizara et al., 2012). Identification of core beliefs associated with specific diseases might fruitfully be applied to the study of multiple sclerosis as well.

Table 4 represents a brief comparison of the limiting beliefs and behaviors observed in a convenience sample of clients with MS and clients with other chronic diseases in a single medical hypnotherapy practice. Data on beliefs and behaviors were documented from sessions with more than 60 people diagnosed with MS and 90 people diagnosed with other chronic diseases as part of the clinician’s detailed client notes. Beliefs were identified using the more direct approach of hypnosis and neurolinguistic programming. As this was not a formal study, there was no codified format for recording the information, but rather the clinical hypnotherapist’s client notes were kept according to best clinical practices, recorded with the aim of helping the clinician help the client. Thus, they were as accurate as possible, based on the clinical hypnotherapist’s integrity. This retrospective analysis of existing records was approved by the Institutional Review Board of the California Institute for Human Sciences. The clinician alone was authorized access to these records and was solely responsible for their interpretation. The five categories in the left column of Table 4 represent those beliefs found most commonly and most strongly among people with MS. Client records for people with other chronic diseases were then reviewed for the presence and strengths of these same beliefs. The table presents belief intensity for the most recent 10 clients in each disease category who completed sufficient sessions to gather a full representation of their underlying beliefs. Belief intensity was scored on a 4-point Likert scale.

**Table 4:** Matrix of belief intensity in different chronic diseases.

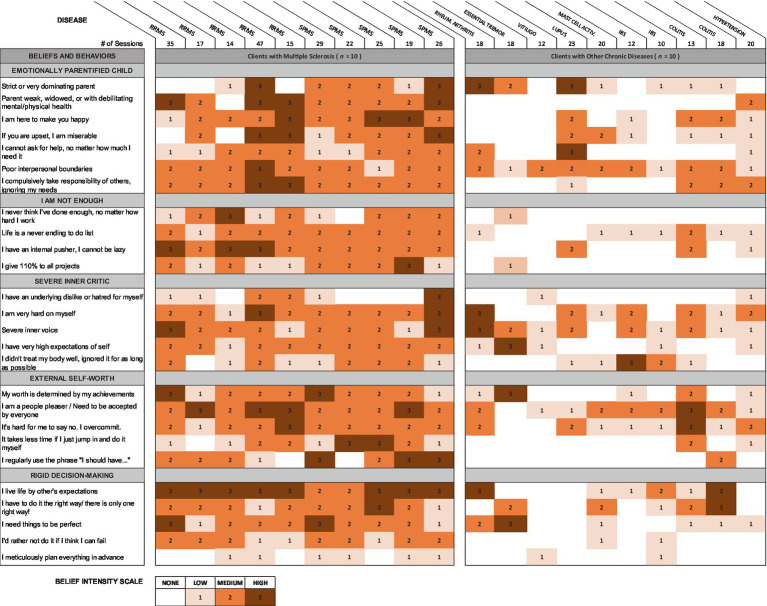

This table supports the idea that while beliefs vary somewhat between people with MS, the presence and intensity of beliefs differ in important ways from people with other chronic diseases. It provides suggestive evidence that the beliefs discussed here might usefully distinguish between people with MS and other chronic diseases. A limitation to these findings is the records were all drawn from a single practice. Further research is needed to more fully identify beliefs common to people with MS and to assess their discriminant ability.

Any one of these categories of limiting beliefs and maladaptive coping behaviors can make situations feel more entrapping, relationship breaches more threatening, and daily life more demanding, significantly contributing to the experience of ongoing stress and increasing allostatic load. A prolonged or unexpected stressor that intensifies the experience of entrapment, threat, and daily demands could provide the physiological impetus to overwhelm the allostatic response system and result in full development and expression of MS.

### Triggering factors

Clinical observation found that people with MS seemed to consistently experience a prolonged period of acute stress along with a more intense relational stressor prior to diagnosis. For some people, symptoms occurred right after these two stressors, for others, they occurred up to 6 months after the issues were resolved. “*Right when I was getting my life back together, I noticed my foot dragging*.” People diagnosed more than 20 years prior did not always recall stressful events prior to onset. However, a deep exploration of that period most often revealed the intense stressor and or culminating relational event. “*Until I started to explore the time before my first symptoms with hypnotherapy, I was completely unaware of how difficult that time period had been for me*.”

The predisposing factors were found to significantly contribute to the degree of stress during this period. In one client, the need to constantly prove herself and to always do even better in order to feel valued, caused her to keep striving even when the circumstances were not favorable. “*There was an economic downturn, but I was determined to increase my sales that year. I never worked so hard in my life.*” While another described how her level of responsibility at work had far surpassed her abilities. Yet, because of her fear of failure, her need to demonstrate her worth, and her belief that asking for help was a weakness, she felt trapped and remained in this bind until disease onset. “*I felt trapped, alone, like the world was on me. By Sunday night my body would shake thinking about returning to work on Monday.*”

In psychology, this combination of lifelong stressors followed by an acute stressor leading to disease development is called the diathesis-stress model (Lazarus, 1993; Monroe and Cummins, 2014). The diathesis (Greek for disposition) could take the form of a biological or genetic vulnerability, early traumatic experiences, or other sociocultural factors. The diathesis-stress model suggests the person will develop a disorder when the combination of the existing predisposition and a current stressor exceed a critical threshold. In the case of MS, the coping behaviors and beliefs learned in response to early childhood developmental challenges create the psychological diathesis and the resulting allostatic load create the physiological diathesis, which together make it more likely the person will develop the disease in response to a stressor in adulthood. This can help explain why one person develops MS following a stressful event while another person exposed to the same stressor does not. Researchers looking at functional neurological disorder, which is similar to MS in many ways, recommend focusing on the stress-diathesis model, suggesting “biological susceptibility interacts with early life adversity, precipitating the disorder by traumatic events later in life and maintained by psychological responses” (Keynejad et al., 2019).

A large-scale study measuring changes in allostatic load in response to similar stressors found people who had experienced more adverse events in childhood showed higher increases in their physiological stress response (Dich et al., 2015). Another large-scale meta-analysis looked at brain function in response to repeated stressors over the lifetime. They found those exposed to repeated or ongoing stress showed heightened emotional reactivity and reduced ability to cope with new stressors (Hosseini-Kamkar et al., 2023). This dynamic of increased physical and emotional reactivity, combined with reduced ability to manage new situations, applies to all stress related factors identified in the triggering factors here and the reinforcing factors in the next section.

#### Prolonged period of stress prior to onset

With rare exceptions, clinical observations recorded a prolonged period of highly intense stress lasting from a few months to a few years prior to disease onset. This acute stressor was experienced as an intense and prolonged feeling of being stuck or trapped. Patients described it as “*I want to get out of this, but I don’t know how, so I push forward*.” This double bind translates into the inability to quit an impossible job situation, or a toxic relationship, and, instead, work harder at it. One client described it as, “*I wanted to leave my job, but I couldn’t as I would lose my work visa*,” while another was so engrained in her habitual pattern to push forward that she had no awareness of her desire to stop. “*I spent almost 14 months working 14-hour days not knowing if they were going to fire me. It never occurred to me to quit*.”

Studies using measures of emotional response to stress consistently find highly stressful events occurring within the 2 years prior to disease onset (Briones-Buixassa et al., 2015). A qualitative study of people’s experiences prior to MS diagnosis reported they showed a slow realization they were in a situation where they were given increasing responsibilities they could not manage, or in which they feared they were inadequate and would fail (Mei-Tal et al., 1970). The tendency to feel stuck and unable to come up with alternative solutions is also supported by research into brain functioning, which found constant stress can lead to reduced functioning of the prefrontal cortex, reducing executive function, while increasing activity in the brain’s emotional centers (Arnsten et al., 2023).

These patterns of increased stressful situations combined with the problematic beliefs and behaviors found in the predisposing factors often lead to significant mental health challenges. A recent large-scale study found people who developed MS experienced mental health disorders in the 5 years prior to diagnosis at much higher rates than the general population (28.0 and 14.9% respectively), which included notably higher rates of depression and anxiety. The disparity between the two groups increased in each of the years getting closer to diagnosis (Chertcoff et al., 2023), which is also consistent with the idea of stressful events preceding onset.

#### Culminating incident

Apart from a prolonged period of stress, clinical observations also identified a culminating incident in many cases, a sort of last straw that was not always directly related to the prolonged stressor. The additional stressor tended to be a relational separation conflict, such as “*We already had the date set, but I realized I couldn’t marry him*.**”** This may include a strong feeling of rejection, “*I confided in my mother and she shocked me telling me how ungrateful I was*,” or a fear they will hurt a loved one, “*I realized my mother would be distraught when I move to college. Was I making the right choice?*”

The response to the threat of a break in an adult relationship echoes in some ways the infant’s response to a break in the caregiver/infant mirroring exercise, where the infant may lose all muscle control (Tronick et al., 1978; Mesman et al., 2009). A psychological stressor that is perceived as unexpected, isolating, and beyond the person’s current ability to respond, can trigger the body to activate its threat response system. When fight or flight have been ruled out, the body switches to the mammalian “freeze” response, physical immobility (Kozlowska et al., 2015). People with a history of difficult experiences show larger reductions in body mobility in response to new perceived threats (Hagenaars et al., 2012). The muscular freezing response is primed by previous experience. People with a history of extreme stress literally lose the ability to move their body in response to a new stressor (Fragkaki et al., 2016). This was found in response to social as well as physical threats (Roelofs et al., 2010), and is larger in people with anxiety (Schmidt et al., 2008). The freeze response can appear as fatigue, blurred vision, weakness or loss of movement in limbs, muscle stiffness, issues with systems controlled by autonomic muscles such as urinary incontinence, and slurred speech, all of which are symptoms of MS (Ghasemi et al., 2017; Lloyd et al., 2019).

The combination of interpersonal conflict in conjunction with a prolonged period of stress or change prior to MS diagnosis was reported in the Mei-Tal et al. case study series 60 years ago (Mei-Tal et al., 1970). Forty years ago Grant et al. published a study using counts of life events graded by emotional intensity, finding about 75% of people had experienced significant difficulty involving a spouse, parent, or sibling shortly prior to onset (Grant et al., 1989). Another study looking at stress prior to MS onset interviewed people many years after their diagnosis (Pratt, 1951). It reported much lower rates of stressors preceding onset. Grant et al. proposed the difference was most probably due to retrospective distortion and loss of memory of those events (Grant et al., 1989). This suggestion is supported by a recent long-term cohort study which found adverse events are likely underreported when asked about many years later (Gehred et al., 2021).

In both the Mei-Tal et al. (1970) and the Grant et al. (1989) studies reported above, the interpersonal stressor incidents were followed by initial symptoms either immediately or within the following 6 months. The delay between events and symptoms mimics that found in delayed-onset PTSD, which may manifest up to 6 months or more after the triggering incident (Horesh et al., 2011). These cases of delayed onset are found more commonly among people who have a history of intense stressful or traumatic events (Andrews et al., 2007; Utzon-Frank et al., 2014).

### Reinforcing factors

Clinical observation found that the diagnosis of multiple sclerosis, with its poor prognosis and subsequent worsening of symptoms, introduces a new set of stressors. Again, these stressors were managed with the same set of maladaptive beliefs and behaviors used previously, adding to the mental and physical stress loads and possibly contributing to disease progression. Two common stressors post-diagnosis were the belief “*I’ll be stuck in a wheelchair*” and the fear of becoming dependent on other people for daily tasks. While disability can be challenging for anyone, having the belief, “*it’s not safe to ask for help*,” the fear of not being able to perform well, and getting “stuck” once again seemed to significantly increase the stress response regarding disability.

Additionally, disease prognosis challenged their definition of self, sense of value in the world, and their vision of the future. “*I am a caregiver turned reluctant patient*.” “*Who is going to want me when I’m just a sack of potatoes?*” “*I had been (in my career) for almost 25 years. I had not trained for nor envisioned any other career. I was lost.*” The prognosis also created a sense of urgency, “*I want to get as much done as I can before MS takes my life away*.” Many people attempted to avoid progression by trying to eat the “right diet” and exercise “enough,” dedicating much of their time to trying to reverse their disease using the same maladaptive coping strategies. “*I feel like I dedicate every moment of my life to trying to get better. It’s exhausting, but I can’t stop*.”

One significant aspect of diagnosis can be found woven throughout the Reinforcing Factors process. Diagnosis of MS presents a threat to the self-identity developed in childhood and usually maintained through life. The threat to identity may lead to change in self-concept (Emery et al., 2022). The changes in identity can be pervasive (Emery et al., 2023) and difficult to navigate (Kiropoulos et al., 2021). The disruption of identity and view of how the world works is a stronger predictor of depression, general anxiety, and stressor-specific anxiety than is the stressful event itself (Milman et al., 2020).

#### Response to diagnosis

Exploration of the day of the diagnosis revealed many clients had a highly acute and distressing experience. “*I thought my life was over.*” The majority of clients did not reach out to others and instead reported dealing with MS for years with only a select few knowing their diagnosis. While a majority reported an increase in stress and depression post-diagnosis, some clients even contemplated suicide. “*I thought about just driving off the road*” and “*I decided that, when these symptoms worsen, I am going to travel to a state where euthanasia is legal*.”

One coping strategy applied by many clients was avoidance. “*I ignored the diagnosis for as long as I could*.” It was observed that this strategy often caused shock and flashbacks of the day of the diagnosis when symptoms did return, however. “*Oh no, it’s back!*” and “*My doctor was right, I am going to end up in a wheelchair!*” were common responses to a marked recurrence of symptoms.

Diagnosis dramatically increases rates of depression and anxiety in people with MS from a rate that was already much higher than the general population (Hoang et al., 2016). Approximately two-thirds of people recently diagnosed with MS are likely to experience hopelessness (Sainz de la Maza et al., 2023), and this prevalence increases as the disease progresses (Patten and Metz, 2002). Hopelessness is independently associated with poorer ability to cope with the disease and a lower quality of life (Patten and Metz, 2002). Hopelessness may also contribute to blood–brain barrier disruption, decreased autonomic nervous system function, impaired decision-making, and neurobehavioral aberrations (Luca et al., 2019). A grounded theory analysis of the stages people move through from medical trauma to growth and healing calls this stage “Diagnosis and Devastation” (Salick and Auerbach, 2006). A meta-analysis of qualitative studies of people reporting their experiences following an MS diagnosis show they consistently go through all five stages of grief associated with loss, making it difficult for them to understand their diagnosis and prognosis (Topcu et al., 2023).

A higher risk of suicide is observed in patients diagnosed with neurological disorders (Alejos et al., 2023). The suicide rate in people recently diagnosed with MS is roughly twice the rate found in the general population (Feinstein and Pavisian, 2017). Up to 65% of people with MS would consider physician-assisted death in the case of severe symptoms such as unbearable pain (Marrie et al., 2017).

An additional contributor of stress post diagnosis is a lack of social support. A study on the relationship of psychosocial factors and MS found that MS patients typically lack external social resources and did not use social support when under stress (Liu et al., 2009). In fact, people with MS make successively less use of social support the longer they have been diagnosed, regardless of disease progression (Lorefice et al., 2018), and tend to use more emotion-oriented coping methods than instrumental methods when the disease does progress (Montel and Bungener, 2007). Although some people with MS use a range of coping strategies (Kotas et al., 2021), avoidant coping strategies are much more common and are associated with increasing depression (Goretti et al., 2009; Lorefice et al., 2018). Both emotion-oriented and avoidant coping strategies are associated with decreases in health-related quality of life in people with MS (Wilski et al., 2019).

#### Beliefs regarding multiple sclerosis

The greatest cause of stress post diagnosis in clinical observation was the negative expectations regarding disease prognosis by the client, their physicians, and society. *“This is a progressive disease. It’s only going to get worse.*” Any new or worsening symptom could trigger a feedback loop in which increased symptoms create more fear of the disease progressing, leading to increased use of maladaptive coping mechanisms, leading to increased allostatic load and further worsening of symptoms. Thus, an increase in symptoms due to injury, flu, vaccination, or overexertion are thought of in terms of disease progression and not understood as the consequence of these additional allostatic loads. *“After the shot, my symptoms got worse for four days. I could barely walk. I was sure I would not recover.”*

Most patients and even physicians hold the view that MS is a “relentlessly progressive, inevitably disabling disease” (Rolak, 2003). A report in 2003 showed only about 20% of patients become bedridden or institutionalized, and another 20% will need some form of assistance such as a cane, walker, or wheelchair. Fully 60% will not require assistance or experience disability. In half of this last group, the disease does not progress and the person experiences only periodic episodes of symptoms (Rolak, 2003). Overall prognosis has continued to improve in the last 25 years with the use of disease-modifying therapies (Koch-Henriksen and Magyari, 2021).

Yet people with MS continue to view their disease as being much more malignant than shown by medical evidence (Heesen et al., 2010; Dennison et al., 2016). This matches the general public perception that the disease is much more rapidly debilitating than shown by medical evidence (Dennison et al., 2016). The negative perception of short-term and long-term consequences of an MS diagnosis is significantly related to anxiety, depression and disease-related distress (Janssens et al., 2004). Anxiety regarding disease progression may increase disability even in the absence of other contributors (Hanna and Strober, 2020). A study using the diathesis-stress model for recording pain and disability after major surgeries found exactly this process taking place, leading to the very real worsening of mental and physical health symptoms (Martin et al., 2010).

Apart from increasing stress, receiving a diagnosis with a difficult prognosis can lead to the patient fulfilling a negative disease course that might not otherwise have occurred (Lamont and Christakis, 2003). Negative beliefs regarding prognosis can produce a worsening of the disease. The placebo effect, where benefit occurs in the absence of a known effective treatment, is recognized by most (Beecher, 1955), but the majority of physicians and patients are not familiar with the nocebo effect, in which harm occurs in the absence of a noxious agent (Kennedy, 1961; Krefting et al., 2023). The nocebo effect can produce new symptoms or worsen existing ones simply through verbal or non-verbal doctor-patient communications (Häuser et al., 2012), or by seeing others with the same disease experience worsening symptoms (Webster et al., 2016). Such negative effects are most commonly found among people with higher anxiety or psychological distress, or have a history of medically unexplained symptoms (Colloca and Barsky, 2020), all of which are common among people diagnosed with MS (Hoang et al., 2016).

The fear of symptoms getting worse and the uncertainty of their progression is a significant source of psychological distress in patients with MS (Mullins et al., 2001). This anxiety regarding the course of illness is thought to cause patients to become hypervigilant regarding their symptoms, resulting in an amplified perception of benign sensations and physical symptoms (Barsky, 1992). Pain catastrophizing, for example, is common in people with MS, and contributes to greater pain interference (Harrison et al., 2015).

#### Living with symptoms of MS

Ongoing symptoms are expected in primary progressive forms of MS, but many people with relapsing–remitting forms also experience symptoms on a daily basis, though less consistently. “*Sometimes I feel almost normal and other days I can barely lift myself out of bed and have to cancel all my plans*.” Clinical observation found that symptoms caused a multitude of daily limitations that increased the experience of stress. Symptoms caused plans to be canceled and dictated the number of activities possible each day. “*If I need to take a shower today, that’s about all I’ll be able to get to toda*y.” Many symptoms of MS are invisible to others, thus the limitations experienced are not apparent or understood by others, including family members, work colleagues, and agencies evaluating disability. “*My husband doesn’t understand how some days I can make us dinner and others days I can’t seem to do anything at all*.” This was especially difficult when the diagnosis is not shared widely. “*My colleagues at work think I’m just trying to get out of working. They don’t know I have MS.*”

Clinical observation found that symptoms would increase temporarily in response to situations, events, or things that are similar to the triggering factors prior to onset. These exacerbations seemed random to the client until they recognized the connection between those types of situations and their body’s response to them. One client who had worked in a highly stressful healthcare environment before onset observed that “*Any time I walk into a hospital, I get all tingly.*” Another immediately felt her body tighten into the “*MS hugs*” when she thinks she might be late. One client, who had taken up smoking while in a significantly stressful relationship a few years prior to onset, found that “*If I smoke a few cigarettes one night, my legs become stiffer by the next morning*.” And, even minor experiences of feeling stuck caused temporary worsening. “*My friend wanted me to go shopping with her this weekend, but I really didn’t want to. I went anyway. The next morning I could barely walk up the stairs!*”

Though most times, new ongoing or acute stressors seem to be followed by a momentary increase in symptoms, occasionally symptoms would not fully recede. Clinical observation suggests this could be due to the intensity and duration of the stressors experienced. “*The fires came right up to our property. I felt so helpless. I never fully recovered my walking*.” These experiences are similar to the triggering factors and could be a key to disease progression.

The quality of life and functional disabilities of people with MS may fluctuate even daily in response to psychological processes. Some increase in physical symptoms was found to follow days with depressed mood (Kasser et al., 2017; Kratz et al., 2019). A study of people living with MS used weekly tracking of stress. It found that stressful events doubled the risk of exacerbations (Buljevac et al., 2003). Polick et al. (2023a) found that more emotionally-salient stress in childhood strongly correlated with the severity of physical symptoms of MS, consistent with the developmental model of core beliefs leading to the expression of the disease. When looking at symptom relapses during COVID however, childhood stressors first showed significance as predictors, but additional analysis found the more recent emotionally-salient stressors, along with use of poor coping behaviors, predicted relapse even more strongly (Polick et al., 2023a).

Invisible symptoms can be the most disturbing and distressing symptoms experienced (White et al., 2008). While the general public is often aware of the visible symptoms such as difficulties with walking and balance, invisible symptoms such as fatigue, pain, anxiety, and depression are not often recognized, yet are disruptive. Significant others, workplaces environments, and even caregivers oftentimes are unaware of symptoms or forget they exist, leading to uncomfortable misunderstandings (Parker et al., 2021). This is similar to the double-bind experienced by people with brain injuries where a person’s level of ability in any given moment is not aligned with other people’s perception of their health (Krefting, 1990).

### Compounding effects of stress in MS

**Figure 2:**
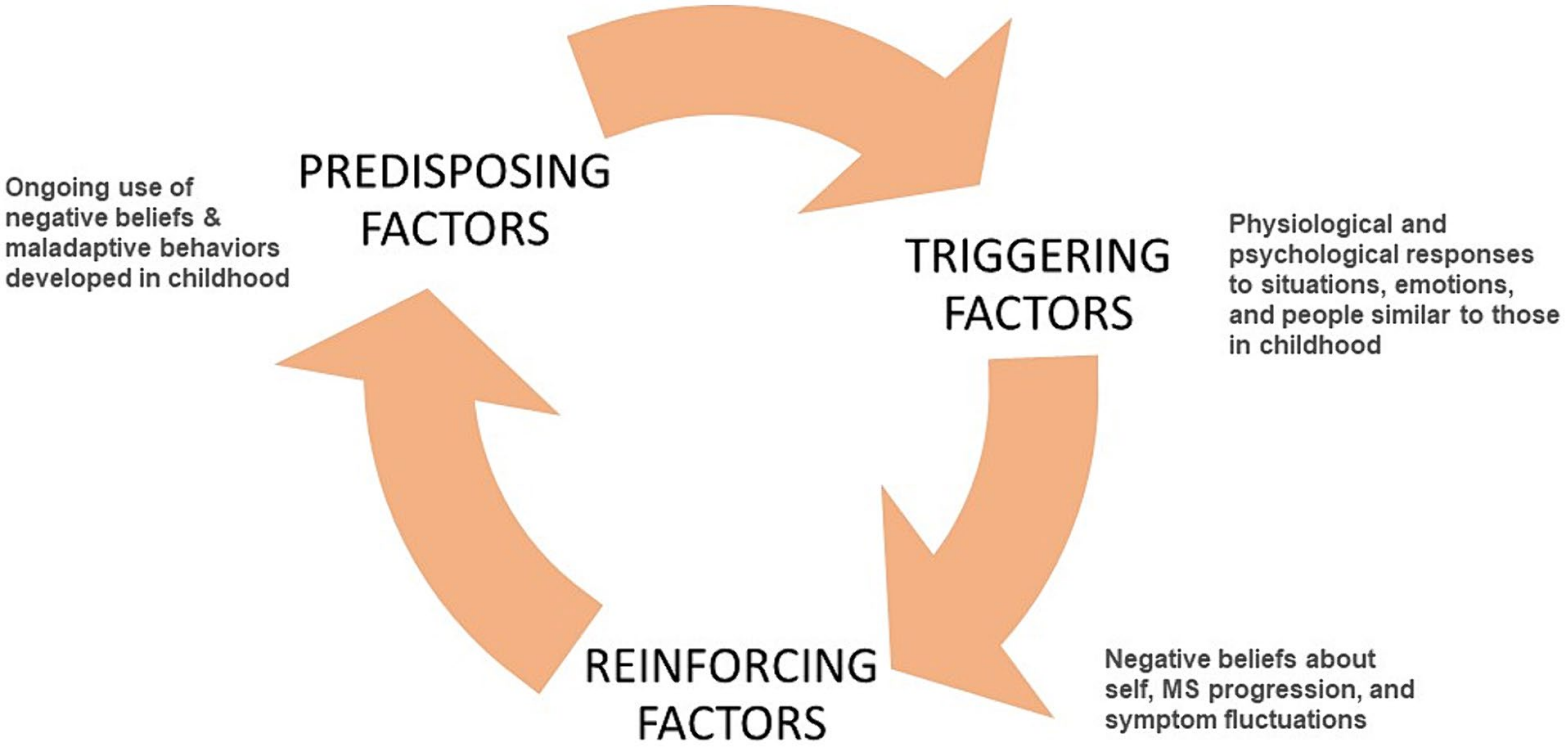
Compounding effects of stress in MS.

These three categories of stress factors create a feedback loop in which each of the stressors build on each other, compounding the effects (Figure 2). The predisposing factors set the stage for distorted threat perception and maladaptive responses. The triggering factors, a combination of acute stressors intensified through the lens of these negative beliefs, cause a more severe experience of feeling stuck, of relationship discord feeling threatening, and of life being more difficult. Then the reinforcing factors add additional stressors that threaten the person’s very identity, and well as highlight the limitations of their existing patterns for coping with difficulty. The persistent beliefs and behaviors developed in childhood are woven throughout all the stages, leading to increasing levels of stress in everyday activities.

Clinical observation found that feedback between beliefs, situations, and symptoms happened continuously. In a recent session, a client with MS reflected on how she does not know how to cancel plans she does not want to do, and she does not know how to not do laundry when she’s too tired. Instead, she just pushes through and does them. Afterward, her body is “*out the next day*.” When the double-bind that caused the experience of being stuck in these two situations was explored, the client exclaimed, *“Oh my gosh, I experience that conflict almost every day!!!”*

Once the pattern of maladaptive core beliefs and behaviors is identified, and the categories of stress factors are understood, their dynamic interplay and expression in people with MS becomes much easier to recognize. A 20 min documentary of a woman living with MS, *Lydia Emily’s Last Mural* (Green, 2018), shows that she pushed herself to continue painting despite agonizing pain and disease progression. She said her first neurologist told her she had only a few years left before MS would take her faculties away. She contemplated assisted suicide in the future when her disease progresses, and said she felt driven to keep working long hours, despite constant pain and progressive impairment. She said it was all worth it because “*It makes people so happy*.”

### A mind-based therapy pilot study

Building on the Developmental Model of Stress and MS, we implemented a pilot study to test a mind-based therapy aimed at treating the causes of stress to treat a disease. The study was reviewed and approved by the Institutional Review Board at the California Institute for Human Science. Briefly, a convenience sample (*n* = 9) was recruited and consented, five with relapsing–remitting MS and four with secondary progressive MS, all women. The novel intervention combined hypnotherapy, neurolinguistic programming, and eye movement integration. These techniques were chosen for their ability to change limiting beliefs and behaviors, build resilience, and restore physical function. Interventions were delivered in-person during 18 private sessions lasting 90–120 min every 2 weeks. Participants were given a hypnosis recording after sessions to listen to between sessions. The MS Quality of Life-54 questionnaire (Vickrey et al., 1995) was administered before the 1st session, and within 2 weeks after the 12th and 18th sessions.

**Figure 3:**
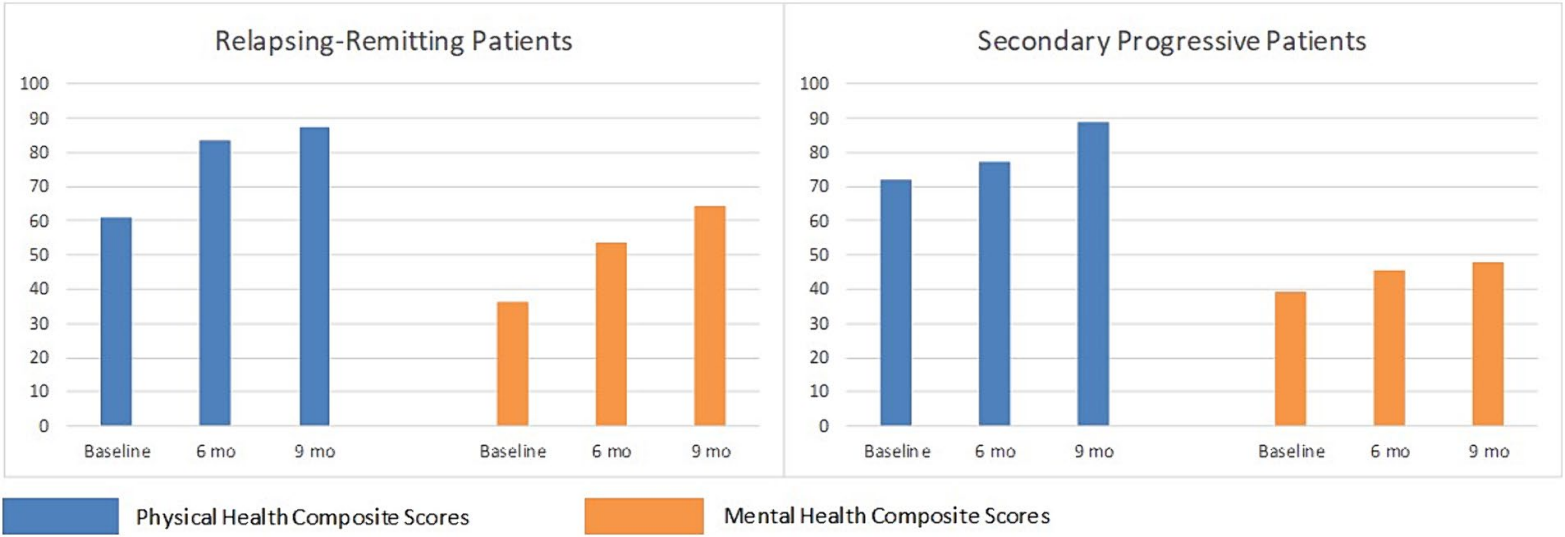
Pilot study results.

Results showed statistically large effect-size improvements in MSQOL-54 scores after 12 sessions, with further improvements after the 18th session. Hedge’s *g* effect sizes were 1.31 for total change in mental health composite scores and 1.28 for total change in physical health composite scores (Figure 3). Mean composite scores in both mental and physical health showed large and clinically important improvement. The results support the hypothesis.

These findings suggest that treating the types of stress associated with MS onset and progression based on the Developmental Model of Stress and MS may produce significant improvement in mental and physical health. The study’s findings suggest the novel multimodal intervention effectively reduced symptoms across multiple domains important to people with MS. More rigorous study is warranted, especially to account for possible confounding or mediating factors, such as genetic, lifestyle, and environmental risk factors (Olsson et al., 2017). If future research yields similar findings, this could lead to a new form of effective MS treatment. For full study details, see Supplementary material.

## Discussion

This paper described the evolution of stress models from Hans Selye’s original Stressor-Stress Response Model to Lazarus’s Cognitive Model of Stress, Engel’s Biopsychosocial Model of Medicine, and Miller et al.’s Biological Embedding of Childhood Adversity. It builds on these models by integrating insights from developmental psychology, expanding the causes of stress from only events to include psychological processes. An extensive body of research in medicine and psychology provides support for this new framework for understanding the role of childhood developmental challenges in creating lifelong experiences of stress that can lead to physical diseases. These childhood experiences form negative core beliefs and behaviors that in turn increase threat perception and maladaptive stress responses. The Developmental Model of Stress proposed here could be applied to many stress-related diseases.

The evolving models of stress also lead to an evolving understanding of how to measure stress and its contributing factors. It moves science forward from first measuring traumatic or adverse events, to then recognizing the importance of the emotional response to events, to now assessing the beliefs and behaviors that give rise to those emotional responses. By doing so, it also opens the door to considering other types of psychosocial stress not tied to traditional models of traumatic events. Implementation of these expanded tools for exploring stress could lead to more coherent interpretations of existing stress-disease research, and potentially to improved study designs for greater precision when exploring the stress-disease relationship. An expanded consideration of what to measure and how could then increase the ability to predict disease development and provide guidelines for more targeted efforts in disease prevention and treatment.

The Developmental Model of Stress and MS applies this evolving understanding to multiple sclerosis specifically. It represents the first attempt to identify negative core beliefs common to people with MS, and map their effects on all other stressors involved in the development and progression of multiple sclerosis. The identified negative beliefs and behaviors may be distinct from those of people diagnosed with other chronic diseases. These particular beliefs and behaviors seem to have been developed during childhood relational challenges, especially relating to emotional needs and autonomy. This creates increased allostatic load, the background buzz of nervous system activation, that increases over the years and creates systemic dysregulation. High allostatic load provides the necessary preconditions for the diathesis-stress model, which shows that the addition of an acute stressor to a weakened or unbalanced system can lead to disease development. For MS, the acute stressor often takes two forms. First a prolonged incident that is experienced as feeling trapped or stuck, and second a threatening relationship discord. This combination of stressors is proposed to contribute to triggering disease onset. The diagnosis of a chronic disease with a poor prognosis and symptom fluctuation adds to the stressors and the body’s stress response, further increasing physiological dysregulation and symptom severity.

Although the potentially causal relationship between stress and MS was first recognized when the disease was named almost 150 years ago, modern research shows conflicting results. These apparent conflicts dissipate when the literature is interpreted using current best practices, strongly suggesting stress as a possible causal factor in the development and progression of MS. This suggestion has profound implications for future research and possible treatment.

Current stress reduction efforts focus mostly on helping people manage the stress of living with a serious disease. Treatment of the underlying beliefs and behaviors could address the cause of stress and potentially influence the course of the disease. Preliminary results of a small pilot study were promising, and suggest that addressing the predisposing, triggering, and reinforcing factors of stress in MS is associated with measurable improvements in physical and mental well-being. Future research to test the Developmental Model of Stress might expand on the pilot study using a more robust sample and design.

## Data availability statement

The datasets used in the content analyses are confidential and thus not able to be shared. Data for the pilot study are available for review. Requests to access the datasets should be directed to EC, eva@evamclark.com.

## Ethics statement

The study involving humans were approved by California Institute for Human Science. The study was conducted in accordance with the local legislation and institutional requirements. The participants provided their written informed consent to participate in this study.

## Author contributions

MF: Conceptualization, Formal analysis, Funding acquisition, Methodology, Supervision, Writing – original draft, Writing – review & editing. EC: Conceptualization, Data curation, Formal analysis, Funding acquisition, Methodology, Visualization, Writing – original draft, Writing – review & editing. CS: Methodology, Supervision, Validation, Visualization, Writing – review & editing.
