# Supplementary material for: A new framework for understanding stress and disease: the developmental model of stress as applied to multiple sclerosis
Source: Front Integr Neurosci. 2024 Jun 17;18:1365672. doi: 10.3389/fnint.2024.1365672 (PMC11218666; doi:10.3389/fnint.2024.1365672)
Supplement: Supplementary file 1 [file Data_Sheet_1.docx]

**Supplemental Text:**

**A Novel Intervention for Stress and Multiple Sclerosis:**

**A Pilot Study Based on the Developmental Model of Stress**

Eva M Clark^1^, Michelle Fauver^2^, and Carolyn E Schwartz^3,4^

1. MINDbased Healing, Santa Cruz, CA, USA
2. Integral Health Program, California Institute for Human Science, Encinitas, CA, USA
3. DeltaQuest Foundation, Inc., Concord, MA, USA
4. Departments of Medicine and Orthopaedic Surgery, Tufts University Medical School, Boston, MA, USA

# Introduction

Multiple sclerosis (MS) is a demyelinating and neurodegenerative disease of the central nervous system. Most people are diagnosed with the relapsing-remitting form of MS, which includes sudden outbreaks of symptoms followed by partial or full recovery (1). More than half of these will eventually move to the secondary progressive form of MS, in which symptoms worsen without remission or full recovery (1). The symptoms of MS are heterogeneous. The majority of people with MS experience mobility impairments (2) and fatigue (3), and more than a quarter experience depression and anxiety (4, 5). Other symptoms may include sensory disorders, digestive tract and bowel dysfunction, pain, muscle weakness and spasms, as well as issues with learning and memory (1).

Standard medical treatments target inflammation (6). These treatments slow disease progression (7), but do not address other factors contributing to the disease. The cause of MS has not yet been found. Many contributing factors have been identified, but their casual relationships remain unclear (8). Stress is recognized as one of the contributing factors.

Many people report stressful experiences preceding MS onset and exacerbations (9, 10). A high quality cross-sectional analysis of stress across the lifespan found both childhood stressors and adult stressors predicted grade of MS symptom severity, with recent stressors predicting symptom worsening (11). But other studies report conflicting findings, with some saying there is a strong correlation between stress and MS and others saying there is no relationship (12). These discrepancies may come down to the types of stress measured and how they are measured (12-14).

Treatment of stress in MS has focused on stress management, helping people cope with the stress of living with a debilitating and often painful disease (15). A systematic review of the effectiveness of treatments for stress on one key physical symptom of MS drew no conclusion regarding effectiveness, and the only study with follow-up data showed the improvements were not sustained (16). A systematic review of general psychological interventions for people with MS noted greater mental health improvements with more intensive and focused treatments (17). Psychological interventions targeting specific mental health symptoms effectively reduced depression, pain, and anxiety, and have now become first line treatment for MS-related fatigue (18-20). Focused treatments also showed improvements in overall physical health, but none of the included studies assessed change in physical disabilities (17).

Yet psychological interventions can produce physical change. Psychotherapeutic treatments have been found to reduce or and even eliminate physical symptoms in people with debilitating chronic musculoskeletal pain, ischemic coronary heart disease, and irritable bowel syndrome (21-23). These treatments did not focus on stress management or symptom reduction, but instead targeted specific psychological precursors of the diseases. Applying these treatment methods to MS would require detailed knowledge of the relationship between stress and MS.

The article to which this paper serves as a supplement (24) describes the differences between the types of stress reported by people with MS and the types of stress measured in research, which could partially explain the conflicting evidence for the role of stress in MS and why current stress management therapies have shown limited benefit. It proposes the Developmental Model of Stress as part of a new framework for understanding stress and MS.

The developmental model adds insights from developmental psychology on how stress is created (24). Extensive research shows children go through stages of psychosocial development (25, 26). These stages focus on developing a healthy sense of self and learning how to build healthy relationships (27-31). When the stages are not completed successfully due to challenges in the parent-child relationship, the child can develop negative core beliefs about themselves and the world around them (32). The negative core beliefs unconsciously influence their stress perceptions and responses (33), leading them to experience more threating events than others with healthy core beliefs and respond to them less effectively (33, 34). The patterns of distorted stress perception and response continue to build and unconsciously influence their mental health and relationships into adulthood (35, 36).

These maladaptive patterns create ongoing and increasing stress, increasing the body’s stress responses (37). This heightened physiological activation is common in people with MS (38). The increased physiological response to stress has been found to produce many of the symptoms of MS. Not just the nervous system inflammation targeted by current pharmaceutical disease-modifying therapies (39, 40), but also neurodegeneration (41), and motor dysfunction (42). It can produce chronic pain (43), emotional dysregulation (43-46), and increase disability (39).

Treating the developmental causes of stress means treating the complex combination of long-established and unconscious core beliefs and coping patterns. Research shows these complex patterns are not well-addressed through conventional psychotherapy and require working with the unconscious negative core beliefs themselves (47). The most effective approaches for people with MS combine hypnotherapy with techniques to address and change the beliefs (48).

The Developmental Model of Stress provides the foundation for a new framework for understanding stress and MS (24). The new framework identifies three categories of stress in people with MS, as shown in Figure 1. The predisposing factors are the combination of maladaptive beliefs and behaviors developed in childhood. There five developmental responses that may be predictive of MS identified in the companion article (24). The triggering factors are the combination of a prolonged period of acute stress and a significant culminating stressor immediately preceding disease onset, with onset occasionally delayed by up to 6 months. The reinforcing factors include being diagnosed with a chronic disease with no known cure, expectations regarding disease progression, and fear of symptoms, with exacerbations and progression influenced by stressful situations similar to those preceding onset.


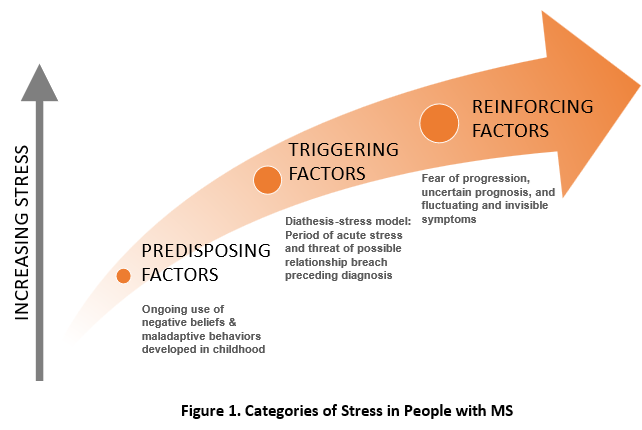


This pilot study sought to treat these three categories of stressors specifically rather than managing their consequences. The hypothesis was that identifying and changing the maladaptive beliefs and behaviors that give rise to stress would lead to improvements in mental and physical health, as measured by the Multiple Sclerosis Quality of Life scale (49). The pilot study used a multimodal intervention to resolve these factors at the level they were created and became automatic, that is, through working with the unconscious mind (50).

# Method

This intervention used a within-subjects design in which all participants received treatment. There was no comparison group.

## Participants

Participants were a convenience sample of nine people with MS recruited from the Santa Cruz Yoga for MS community and the MS Quality of Life Project in Monterey, both in California. Participants were not paid for their participation, though they did receive free treatment. All participants provided written informed consent and a letter of approval from their neurologist prior to enrollment. The California Institute for Human Science Institutional Review Board reviewed and approved the analysis of existing data.

The study sample was all female with an average age of 60 (range 40-81). The diagnoses were evenly distributed between relapsing-remitting MS and secondary progressive MS. The average time since diagnosis was 20 years (range 6-37). About half had no ambulation disability, and half relied on an assistive device. Table 1 describes the sample demographics more fully.

| **Table 1. Demographic Characteristics of Study Sample at Baseline (*n* = 9)** | | | |
| --- | --- | --- | --- |
| **Characteristic** |  |  |  |
| No. female | 9 |  |  |
| Mean age (SD) | 60 | (13) |  |
| range | 40-81 |  |  |
| Marital Status |  |  |  |
| Single | 2 |  |  |
| Married | 5 |  |  |
| Separated / Divorced | 2 |  |  |
| MS Disease Course |  |  |  |
| Relapsing / Remitting | 4 |  |  |
| Secondary Progressive | 5 |  |  |
| Mean Years Since Diagnosis (SD) | 20 | (10) |  |
| Range | 6-37 |  |  |
| Level of Ambulation Disability at Baseline |  |  |  |
| No disability | 4 |  |  |
| Unilateral Support | 1 |  |  |
| Bilateral Support | 1 |  |  |
| Scooter / Wheelchair | 3 |  |  |

## Intervention

Each participant received 18 private, in-person sessions lasting 90-120 minutes each. Sessions were led by a single experienced practitioner every other week for approximately 9 months. Participants were asked to practice the tools learned and to listen to individualized hypnosis records between sessions. This time frame was designed 1) to gradually integrate changes into daily life, and 2) to allow them to listen to hypnosis recordings between sessions to create lasting belief change as well as stimulate physical changes. Additionally, participants maintained a daily tracking of symptom goals, diet, and exercise. The tracked information was used to guide intervention discussions.


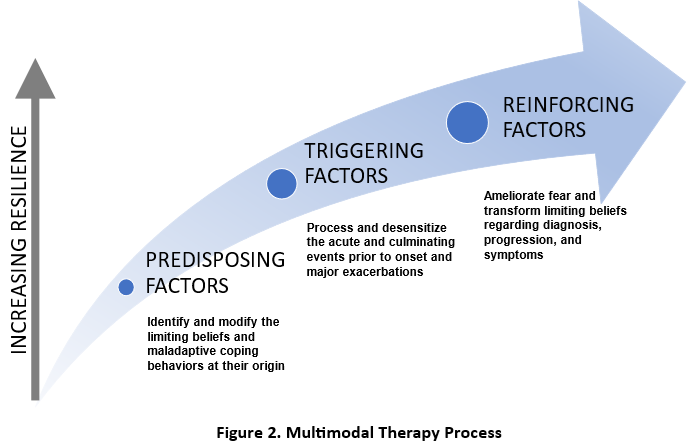


Treatments incorporated hypnotherapy, neurolinguistic programming, and eye movement integration to address the specific stressors defined in the Developmental Model of Stress and MS (24), as outlined in Figure 2. The modalities used to address these stressors were selected for their documented effectiveness in 1) identifying core beliefs and their origins, and 2) producing the desired changes relatively quickly and at the unconscious level where change can be sustained and become automatic.

### Hypnotherapy

Hypnotherapy is beneficial for stress reduction (51), belief and behavioral change (52), and managing symptoms of MS (53). Several methods of hypnosis were applied. Parts therapy in hypnosis was used to talk to internal parts, such as the disease and symptoms, and to understand the limiting beliefs and behaviors associated with them (54). Hypnoanalysis, also referred to as regression therapy, was used to explore the earliest memories related to current emotions, core beliefs, and maladaptive behaviors (55). The process involves 1) identifying a negative emotion, belief, or behavior triggered by an event or symptom, 2) regressing the person to the earliest memory of that same emotion, belief, or behavior (55, 56), which is usually before the age of five, 3) transforming the perception and experience of the past events, and 4) providing greater resilience and resources in the present and the future (57). Hypnotic suggestions, also referred to as guided imagery, were used to stimulate physical recovery, increase effectiveness, and extend the duration of changes created by the hypnoanalysis (22, 52, 58). Additionally, hypnotic suggestion was selected for its effectiveness in creating rapid change in attitude and beliefs (52), decreasing symptoms (53), and restoring function (53, 59, 60).

Hypnosis recordings were created during the sessions for participants to listen to at home. These recordings helped to embed belief change at the subconscious level, stimulate physical change (61), and experience a future self that practices greater resilience and adaptive behaviors (62). The recordings used both direct and indirect suggestions, as the combination has been proven to be more effective in creating physical change than each alone (63, 64).

### Neurolinguistic Programming

Research on neurolinguistic programming (NLP) is still in its infancy, and many studies are inconclusive. A comprehensive review noted that most NLP studies are not done with experienced practitioners, and the majority test principles of NLP rather than actual treatments (65). A set of studies that did use full treatments looked at its effects on complex post-traumatic stress in veterans (66-68). These studies found significant symptom improvement, with more than 90% of participants who completed treatment experiencing complete remission.

NLP was used in this pilot study to modify systemic beliefs and behaviors (52, 69-71), reduce anxiety and stress (69), and improve coping behaviors (69, 72). As with hypnoanalysis, NLP was also used to discover core beliefs and the initial sensitizing events that contributed to their formation (73).

Specific NLP techniques were used to modify the inner critic (74), eliminate guilt and shame (74), reduce the fear of disease progression and symptoms, learn to be internally referenced (75), build stronger boundaries (76), and increase resilience and resourcefulness. The counterexample process (77) was applied to reduce the conditioned “freeze” response to experiences with similar feelings of being trapped or stuck as the initial triggering events that occurred prior to MS onset.

### Eye Movement Integration

Eye movement integration (EMI) uses guided eye movements to reduce the negative emotional charge connected to adverse experiences (78), including those of childhood adversity (79, 80). EMI was used in the pilot as the first step in letting go of the negative emotions associated with the person’s experiences prior to disease onset and symptom exacerbations. It was also used to help break down limiting beliefs, behaviors, and irrational fears of symptoms and progression for later emotional processing (78, 81-83).

## Outcome Measures

The MS Quality of Life Instrument-54 (MSQOL-54) was the primary outcome measure, with administrations at baseline, within 2 weeks after the 12th session (6 months), and again within 2 weeks after the 18th session (9 months). This 54-item self-report measure has satisfactory validity and reliability (49). The MSQOL-54 is summarized with two composite scores reflecting physical and mental health. Statistical analyses were done only on these two composite scores.

## Statistical Analysis

The small sample size cannot be assumed to display a normal distribution, so non-parametric tests of significance were used. The Friedman test of differences among repeated measures for the Mental Health Composite Scores and Physical Health Composite scores both showed statistical significance below *p* = 0.05, but are not being reported here as *p*-values provide little to no information of value (84). Especially for clinical trials, effect size is much more important. Cohen’s *d* is viewed as the best statistic for reporting effect sizes (85). This analysis used Hedge’s *g* instead. Hedges *g* is more accurate for small sample sizes than Cohen’s *d* because it uses sample variance instead of population variance (86).

# Results

Results revealed statistically very large effect-size improvements in MSQOL-54 scores over 12- and 18-sessions of follow-up (Figure 3). The Hedge’s *g* effect sizes were 1.31 for total change in mental health composite scores and 1.28 for total change in physical health composite scores. As with Cohen’s *d*, a change of 0.2 is considered small, 0.5 is medium, and 0.8 is large. Mental and physical health scores improved for people with relapsing remitting MS, secondary progressive MS, for those recently diagnosed, and those with more than 30 years of debilitating symptoms.


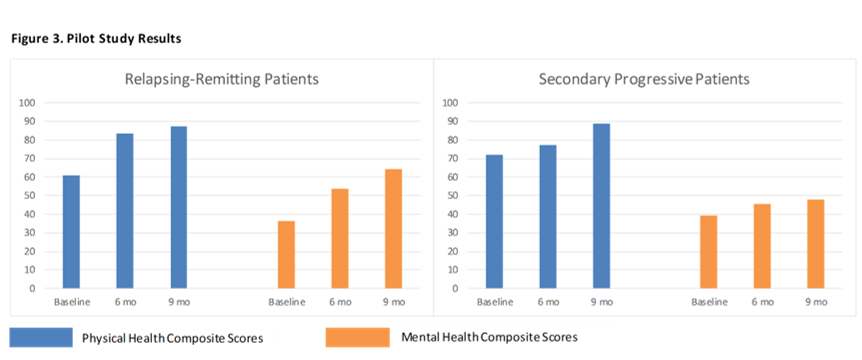


The minimum clinically important difference is smallest amount of change the person is able to recognize and appreciate. This has been found to be approximately one half of the standard deviation for health-related quality of life scales (87). The MSQOL composite scores have a mean of 50 and standard deviation of 10 (49), so a change of 5 points is clinically important. Overall, the physical health composite score means were 39 at baseline, 49 at 6 months, and 57 at 9 months, for a total improvement of 18 points. The mental health composite score means were 67 points at baseline, 80 at 6 months, and 88 at 9 months, for a total improvement of 21 points. Especially large score improvements were found in the cognitive function, energy, and pain subscales.

# Discussion

Most stress-related interventions for people with MS are designed to treat the stress of living with a chronic and progressively debilitating disease. This study is the first to treat the potential causes of stress that may lead to disease onset and progression. It found that the use of a novel intervention combining hypnotherapy, neurolinguistic programming, and eye movement integration to change the three types of stress identified in the Developmental Model of Stress and MS produced significant improvements in physical and mental health. This is the first treatment to show significant improvements across a wide range of MS symptoms.

## Clinical Observations

The treatment was well tolerated despite its relatively demanding frequency and duration. The length of the intervention, while not standard in medical research on psychosocial interventions, facilitated more consistency in improvements and to disease symptoms becoming less responsive to stressful life events. Most people showed significant improvement after the first few sessions, then a small reduction in improvements, and then further improvements after the 9th session. Stressful life events, such as family conflicts, accidents, flu, and holidays, significantly affected symptoms during the first 6 months and less so afterward.

Friends and medical professionals in the participants’ lives reported improvements to the participants that they didn’t recognize themselves. Participants had not noted these improvements when recording in their daily tracking forms. Daily tracking was considered the least accurate measure of change. The improvements observed by others were also not fully reflected in scores on the MS Quality of Life measure, which is another form of self-report. The reduced recognition of change may be due to response shift, in which the person shifts attention from the positive changes to the problems that remain (88, 89).

## Limitations

This pilot study provided an opportunity to evaluate the impact of a novel intervention targeting the psychological stress precursors of MS on the symptoms of MS. As no control group was used, the results must be interpreted with caution. Competing explanations for the substantial improvements might include the placebo effect (90) and selection bias (91). Treatment was delivered by a single provider. Although individually the techniques are widely used, the combination of techniques used here are not. More research is needed to see if the provided care can be effectively delivered by others. The outcomes measured were relatively broad (i.e., physical and mental health) because the sample was too small to report on specific symptom domains. Additionally, while the MSQOL-54 is moderately responsive to changes in physical and mental health, it does not provide sufficiently detailed information about symptoms affected by the intervention. The study does not test the accuracy of the Developmental Model of Stress and MS, nor whether the novel intervention produces the types of change intended. It shows only that the results obtained are consistent with the predictions of the model and the intentions of the treatment.

## Conclusions

Treating the types of stress associated the MS onset and progression based on the Developmental Model of Stress and MS may produce significant improvement in mental and physical health. The study’s findings suggest the novel multimodal intervention effectively reduced MS symptoms across multiple domains important to patients.

This study’s positive results warrant future research with a more rigorous clinical trial design, treatment delivered by multiple providers, and a broader range of outcome measures more accurately responsive to change. Future research might also evaluate whether the novel treatment produces the intended changes in beliefs and behaviors, and to identify possible additional stress factors beyond those named in the Developmental Model of Stress and MS. If the pilot study’s findings are supported, it could open the door to a new category of effective treatments for MS.

**Acknowledgements.** The authors wish to thank Grant Helm, Executive Director of the Multiple Sclerosis Quality of Life Project, for facilitating participant recruitment and procuring funding for this study. San Francisco chapter of the National Multiple Sclerosis Society provided partial funding of the pilot study. We are grateful to the California Institute for Human Science for use of their Institutional Review Board and for paying the publication costs.

# References

1. Ghasemi N, Razavi S, Nikzad E. Multiple sclerosis: Pathogenesis, symptoms, diagnoses and cell-based therapy. Cell Journal (Yakhteh). 2017;19(1):1.

2. Sutliff MH. Contribution of impaired mobility to patient burden in multiple sclerosis. Current Medical Research and Opinion. 2010;26(1):109-19.

3. Oliva Ramirez A, Keenan A, Kalau O, Worthington E, Cohen L, Singh S. Prevalence and burden of multiple sclerosis-related fatigue: A systematic literature review. BMC Neurology. 2021;21.

4. Marrie RA, McFadyen C, Yaeger L, Salter A. A systematic review of the validity and reliability of the Patient-Determined Disease Steps scale. International Journal of MS Care. 2023;25(1):20-5.

5. Boeschoten RE, Braamse AM, Beekman AT, Cuijpers P, van Oppen P, Dekker J, et al. Prevalence of depression and anxiety in multiple sclerosis: A systematic review and meta-analysis. Journal of the Neurological Sciences. 2017;372:331-41.

6. Vargas DL, Tyor WR. Update on disease-modifying therapies for multiple sclerosis. Journal of Investigative Medicine. 2017;65(5):883-91.

7. Bebo Jr BF, Allegretta M, Landsman D, Zackowski KM, Brabazon F, Kostich WA, et al. Pathways to cures for multiple sclerosis: A research roadmap. Multiple Sclerosis Journal. 2022;28(3):331-45.

8. Zarghami A, Li Y, Claflin SB, van der Mei I, Taylor BV. Role of environmental factors in multiple sclerosis. Expert Review of Neurotherapeutics. 2021;21(12):1389-408.

9. Mohr DC, Bhattarai J. Talking with your patients about difficult topics: Stress. National Multiple Sclerosis Society; 2018.

10. Mei-Tal V, Meyerowitz S, Engel GL. The role of psychological process in a somatic disorder: Multiple sclerosis. 1. The emotional setting of illness onset and exacerbation. Psychosomatic Medicine. 1970;32(1):67-86.

11. Polick CS, Ploutz-Snyder R, Braley TJ, Connell CM, Stoddard SA. Associations among stressors across the lifespan, disability, and relapses in adults with multiple sclerosis. Brain and Behavior. 2023;13(7):e3073.

12. Briones-Buixassa L, Milà R, Mª Aragonès J, Bufill E, Olaya B, Arrufat FX. Stress and multiple sclerosis: A systematic review considering potential moderating and mediating factors and methods of assessing stress. Health Psychology Open. 2015;2(2).

13. Koolhaas JM, Bartolomucci A, Buwalda B, de Boer SF, Flügge G, Korte SM, et al. Stress revisited: A critical evaluation of the stress concept. Neuroscience & Biobehavioral Reviews. 2011;35(5):1291-301.

14. Shields GS, Fassett‐Carman A, Gray ZJ, Gonzales JE, Snyder HR, Slavich GM. Why is subjective stress severity a stronger predictor of health than stressor exposure? A preregistered two‐study test of two hypotheses. Stress and Health. 2023;39(1):87-102.

15. Ben‐Zacharia AB. Therapeutics for multiple sclerosis symptoms. Mount Sinai Journal of Medicine: A Journal of Translational and Personalized Medicine. 2011;78(2):176-91.

16. Mohr DC, Lovera J, Brown T, Cohen B, Neylan T, Henry R, et al. A randomized trial of stress management for the prevention of new brain lesions in MS. Neurology. 2012;79(5):412-9.

17. Pagnini F, Bosma CM, Phillips D, Langer E. Symptom changes in multiple sclerosis following psychological interventions: A systematic review. BMC Neurology. 2014;14(1).

18. Phyo AZZ, Demaneuf T, De Livera AM, Jelinek GA, Brown CR, Marck CH, et al. The efficacy of psychological interventions for managing fatigue in people with multiple sclerosis: A systematic review and meta-analysis. Frontiers in neurology. 2018;9.

19. Hind D, Cotter J, Thake A, Bradburn M, Cooper C, Isaac C, et al. Cognitive behavioural therapy for the treatment of depression in people with multiple sclerosis: A systematic review and meta-analysis. BMC Psychiatry. 2014;14(5):1-13.

20. Montañés‐Masias B, Bort‐Roig J, Pascual JC, Soler J, Briones‐Buixassa L. Online psychological interventions to improve symptoms in multiple sclerosis: A systematic review. Acta Neurologica Scandinavica. 2022;146(5):448-64.

21. Yarns BC, Lumley MA, Cassidy JT, Steers WN, Osato S, Schubiner H, et al. Emotional awareness and expression therapy achieves greater pain reduction than cognitive behavioral therapy in older adults with chronic musculoskeletal pain: A preliminary randomized comparison trial. Pain Medicine. 2020;21(11):2811-22.

22. Carolusson S. Dynamic hypnosis, IBS, and the value of individualizing treatment: A clinical perspective. International Journal of Clinical and Experimental Hypnosis. 2014;62(2):145-63.

23. Ketterer MW, Mahr G, Goldberg AD. Psychological factors affecting a medical condition: Ischemic coronary heart disease. Journal of Psychosomatic Research. 2000;48(4-5):357-67.

24. Fauver M, Clark EM, Schwartz CE. A new framework for understanding stress and disease: The Developmental Model of Stress as applied to multiple sclerosis. Frontiers in Integrative Neuroscience. 2024.

25. Bosma HA, Kunnen ES. Determinants and mechanisms in ego identity development: A review and synthesis. Developmental Review. 2001;21(1):39-66.

26. Vaillant GE, Milofsky E. Natural history of male psychological health: IX. Empirical evidence for Erikson's model of the life cycle. The American journal of psychiatry. 1980;137(11):1348-59.

27. Erikson EH. Childhood and society: Norton; 1950.

28. Erikson EH. Identity: Youth and crisis: Norton; 1968.

29. Erikson EH. Identity and the life cycle. Selected papers.: International Universities Press; 1959.

30. Franz CE, White KM. Individuation and attachment in personality development: Extending Erikson's theory. Journal of Personality. 1985;53(2):224-56.

31. Gilligan C. New maps of development: New visions of maturity. American Journal of Orthopsychiatry. 1982;52(2):199-212.

32. Marcia J, Josselson R. Eriksonian personality research and its implications for psychotherapy. Journal of Personality. 2013;81(6):617-29.

33. Rakhshani A, Furr RM. The reciprocal impacts of adversity and personality traits: A prospective longitudinal study of growth, change, and the power of personality. Journal of Personality. 2021;89(1):50-67.

34. McLaughlin KA, Sheridan MA, Alves S, Mendes WB. Child maltreatment and autonomic nervous system reactivity: Identifying dysregulated stress reactivity patterns using the biopsychosocial model of challenge and threat. Psychosomatic Medicine. 2014;76(7):538-46.

35. Tariq A, Quayle E, Lawrie SM, Reid C, Chan SWY. Relationship between early maladaptive schemas and anxiety in adolescence and young adulthood: A systematic review and meta-analysis. Journal of Affective Disorders. 2021;295:1462-73.

36. McArthur BA, Burke TA, Connolly SL, Olino TM, Lumley MN, Abramson LY, et al. A longitudinal investigation of cognitive self-schemas across adolescent development. Journal of Youth and Adolescence. 2019;48:635-47.

37. Miller GE, Chen E, Parker KJ. Psychological stress in childhood and susceptibility to the chronic diseases of aging: Moving toward a model of behavioral and biological mechanisms. Psychological Bulletin. 2011;137(6):959-97.

38. Waliszewska-Prosół M, Nowakowska-Kotas M, Misiak B, Chojdak-Łukasiewicz J, Budrewicz S, Pokryszko-Dragan A. Allostatic load index in patients with multiple sclerosis: A case-control study. Psychoneuroendocrinology. 2022;142.

39. Gold SM, Mohr DC, Huitinga I, Flachenecker P, Sternberg EM, Heesen C. The role of stress-response systems for the pathogenesis and progression of MS. Trends in Immunology. 2005;26(12):644-52.

40. Lenart-Bugla M, Szcześniak D, Bugla B, Kowalski K, Niwa S, Rymaszewska J, et al. The association between allostatic load and brain: A systematic review. Psychoneuroendocrinology. 2022.

41. Harnett NG, Goodman AM, Knight DC. PTSD-related neuroimaging abnormalities in brain function, structure, and biochemistry. Experimental Neurology. 2020;330.

42. Snell HD, Vitenzon A, Tara E, Chen C, Tindi J, Jordan BA, et al. Mechanism of stress-induced attacks in an episodic neurologic disorder. Science Advances. 2022;8(16).

43. Nakamoto K, Tokuyama S. Stress-induced changes in the endogenous opioid system cause dysfunction of pain and emotion regulation. International Journal of Molecular Sciences. 2023;24(14).

44. Aust S, Härtwig EA, Heuser I, Bajbouj M. The role of early emotional neglect in alexithymia. Psychological Trauma: Theory, Research, Practice, and Policy. 2013;5(3):225-32.

45. Ditzer J, Wong EY, Modi RN, Behnke M, Gross JJ, Talmon A. Child maltreatment and alexithymia: A meta-analytic review. Psychological Bulletin. 2023;149(5-6):311-29.

46. Wearden A, Cook L, Vaughan-Jones J. Adult attachment, alexithymia, symptom reporting, and health-related coping. Journal of Psychosomatic Research. 2003;55(4):341-7.

47. Hawke LD, Provencher MD. Early maladaptive schemas: Relationship with case complexity in mood and anxiety disorders. Journal of Cognitive Psychotherapy. 2013;27(4):359-69.

48. Jensen MP, Ehde DM, Gertz KJ, Stoelb BL, Dillworth TM, Hirsh AT, et al. Effects of self-hypnosis training and cognitive restructuring on daily pain intensity and catastrophizing in individuals with multiple sclerosis and chronic pain. International Journal of Clinical and Experimental Hypnosis. 2010;59(1):45-63.

49. Vickrey BG, Hays RD, Harooni R, Myers LW, Ellison GW. A health-related quality of life measure for multiple sclerosis. Quality of Life Research. 1995;4(3):187-206.

50. Bargh JA, Morsella E. The unconscious mind. Perspectives on Psychological Science. 2008;3(1):73-9.

51. Fisch S, Brinkhaus B, Teut M. Hypnosis in patients with perceived stress: A systematic review. BMC complementary and alternative medicine. 2017;17(1).

52. Van Dessel P, De Houwer J. Hypnotic suggestions can induce rapid change in implicit attitudes. Psychological Science. 2019;30(9):1362-70.

53. Maresca G, Nocito V, Lo Buono V, Latella D, Di Cara M, Formica C, et al. Hypnotherapy as a nonpharmacological treatment for the psychological symptoms of Multiple Sclerosis. Alternative Therapies in Health & Medicine. 2023;29(4):266-9.

54. Hunter R. Hypnosis for inner conflict resolution: Introducing parts therapy: Crown House; 2004.

55. Hunter CR, Eimer BN. The art of hypnotic regression therapy: A clinical guide: Crown House; 2012.

56. Lucas WB. Regression therapy: A handbook for professionals: Transpersonal Publishing; 2007.

57. Mener E, Mener A-C. The E2R (Emotion, regression, repair) method: A case study of this new pragmatic hypnotherapy technique. Complementary Therapies in Clinical Practice. 2023;50.

58. Brambila-Tapia AJL, Gutiérrez-García MM, Ruiz-Sandoval JL, Vázquez-Vázquez D, Ramírez-Martínez JF, Macias-Islas MÁ, et al. Using hypnoanalysis and guided imagery to identify and manage emotional aspects of multiple sclerosis. EXPLORE. 2022;18(1):88-95.

59. De Benedittis G. Hypnosis: From neural mechanisms to clinical practice. OBM Integrative and Complementary Medicine. 2020;5(3):1-7.

60. Pagani M, Högberg G, Fernandez I, Siracusano A. Correlates of EMDR therapy in functional and structural neuroimaging: A critical summary of recent findings. Journal of EMDR Practice and Research. 2013;7(1):29-38.

61. Case LK, Jackson P, Kinkel R, Mills PJ. Guided imagery improves mood, fatigue, and quality of life in individuals with multiple sclerosis: An exploratory efficacy trial of healing light guided imagery. Journal of Evidence-Based Integrative Medicine. 2018;23:2515690X17748744.

62. Yapko MD. Encouraging hindsight in advance: Age progression in therapy–and life. American Journal of Clinical Hypnosis. 2022;65(1):4-17.

63. Dillworth T, Jensen MP. The role of suggestions in hypnosis for chronic pain: A review of the literature. The Open Pain Journal. 2010;3(1):39-51.

64. Thompson T, Terhune DB, Oram C, Sharangparni J, Rouf R, Solmi M, et al. The effectiveness of hypnosis for pain relief: A systematic review and meta-analysis of 85 controlled experimental trials. Neuroscience & Biobehavioral Reviews. 2019;99:298-310.

65. Wake L, Gray R, Bourke F. The clinical effectiveness of neurolinguistic programming: A critical appraisal: Routledge; 2013.

66. Gray R, Budden-Potts D, Bourke F. Reconsolidation of traumatic memories for PTSD: A randomized controlled trial of 74 male veterans. Psychotherapy Research. 2019;29(5):621-39.

67. Gray RM, Bourke F. Remediation of intrusive symptoms of PTSD in fewer than five sessions: a 30-person pre-pilot study of the RTM Protocol. Journal of Military, Veteran and Family Health. 2015;1(2):13-20.

68. Tylee DS, Gray R, Glatt SJ, Bourke F. Evaluation of the reconsolidation of traumatic memories protocol for the treatment of PTSD: A randomized, wait-list-controlled trial. Journal of Military, Veteran and Family Health. 2017;3(1):21-33.

69. Konefal J, Duncan RC. Social anxiety and training in neurolinguistic programming. Psychological Reports. 1998;83(3):1115-22.

70. Davis DI, Davis SL. Belief change and neurolinguistic programming. Family Dynamics of Addiction Quarterly. 1991;1(2):34-44.

71. Dilts R. Changing belief systems with NLP: Meta Publications; 1990.

72. HemmatiMaslakpak M, Farhadi M, Fereidoni J. The effect of neuro-linguistic programming on occupational stress in critical care nurses. Iranian Journal of Nursing and Midwifery Research. 2016;21(1):38-44.

73. James T, Woodsmall W. Time line therapy and the basis of personality: Crown House; 2017.

74. Andreas S. Transforming negative self-talk: Practical, effective exercises: Norton; 2012.

75. Andreas C, Andreas S. Heart of the mind: Engaging your inner power to change with neuro-linguistic programming. Real People Press2018.

76. Andreas S. Transforming your self: Becoming who you want to be: Real People Press; 2003.

77. Dilts R, Hallbom T, Smith S. Beliefs: Pathways to health and well-being: Crown House; 2012.

78. Beaulieu D. An introduction to Eye Movement Integration Therapy. European Journal of Clinical Hypnosis. 2005;6(3):2-11.

79. Struwig E, van Breda AD. An exploratory study on the use of eye movement integration therapy in overcoming childhood trauma. Families in Society. 2012;93(1):29-37.

80. Van Der Spuy C, van Breda AD. An exploratory study on the use of eye movement integration therapy for treating trauma in early childhood in South Africa. Child Care in Practice. 2019;25(2):157-74.

81. Beaulieu D. Eye movement integration therapy: The comprehensive clinical guide: Crown House; 2003.

82. Civilotti C, Cussino M, Callerame C, Fernandez I, Zaccagnino M. Changing the adult state of mind with respect to attachment: An exploratory study of the role of EMDR psychotherapy. Journal of EMDR Practice & Research. 2019;13(3).

83. Deninger M. Multichannel Eye Movement Integration: The brain science path to easy and effective PTSD treatment: Gracie Publications; 2020.

84. Wasserstein RL, Lazar NA. The ASA’s statement on *p*-values: Context, process, and purpose. The American Statistician. 2016;70(2):129-33.

85. Norman GR, Wyrwich KW, Patrick DL. The mathematical relationship among different forms of responsiveness coefficients. Quality of Life Research. 2007;16(5):815-22.

86. Rosnow RL, Rosenthal R. Effect sizes for experimenting psychologists. Canadian Journal of Experimental Psychology/Revue canadienne de psychologie expérimentale. 2003;57(3):221-37.

87. Norman GR, Sloan JA, Wyrwich KW. Interpretation of changes in health-related quality of life: The remarkable universality of half a standard deviation. Medical Care. 2003;41(5):582-92.

88. Schwartz CE, Quaranto BR, Rapkin BD, Healy BC, Vollmer T, Sprangers MAG. Fluctuations in appraisal over time in the context of stable versus non-stable health. Quality of Life Research. 2014;23(1):9-19.

89. Schwartz CE, Huang I-C, Rohde G, Skolasky RL. Listening to the elephant in the room: Response-shift effects in clinical trials research. Journal of Patient-Reported Outcomes. 2022;6(1):105.

90. Beecher HK. The powerful placebo. JAMA: The Journal of the American Medical Association. 1955;159(17):1602-6.

91. Ellenberg JH. Selection bias in observational and experimental studies. Statistics in Medicine. 1994;13(5‐7):557-67.
